# Two-factor authentication underpins the precision of the piRNA pathway

**DOI:** 10.1038/s41586-024-07963-3

**Published:** 2024-09-18

**Authors:** Madeleine Dias Mirandela, Ansgar Zoch, Jessica Leismann, Shaun Webb, Rebecca V. Berrens, Devisree Valsakumar, Yuka Kabayama, Tania Auchynnikava, Martina Schito, Tamoghna Chowdhury, David MacLeod, Xinyu Xiang, Juan Zou, Juri Rappsilber, Robin C. Allshire, Philipp Voigt, Atlanta G. Cook, Joan Barau, Dónal O’Carroll

**Affiliations:** 1 Centre for Regenerative Medicine, Institute for Regeneration and Repair, Institute for Stem Cell Research, University of Edinburgh, Edinburgh, UK; 2 Wellcome Centre for Cell Biology, University of Edinburgh, Edinburgh, UK; 3 Institute of Molecular Biology, Mainz, Germany; 4 IDRM, Department of Paediatrics, University of Oxford, Oxford, UK; 5 Department of Biochemistry, Oxford University, Oxford, UK; 6 Epigenetics Programme, Babraham Institute, Cambridge, UK; 7 Zhejiang University-University of Edinburgh Institute (ZJU-UoE Institute), Zhejiang University School of Medicine, Zhejiang University, Haining, China; 8 Bioanalytics, Institute of Biotechnology, Technische Universität Berlin, Berlin, Germany; 9 Present Address: MRC Human Genetics Unit, Institute of Genetics and Cancer, University of Edinburgh, Western General Hospital, Edinburgh, UK

**Keywords:** Piwi RNAs, Spermatogenesis, DNA methylation

## Abstract

The PIWI-interacting RNA (piRNA) pathway guides the DNA methylation of young, active transposons during germline development in male mice^1^. piRNAs tether the PIWI protein MIWI2 (PIWIL4) to the nascent transposon transcript, resulting in DNA methylation through SPOCD1 (refs. ^2–5^). Transposon methylation requires great precision: every copy needs to be methylated but off-target methylation must be avoided. However, the underlying mechanisms that ensure this precision remain unknown. Here, we show that SPOCD1 interacts directly with SPIN1 (SPINDLIN1), a chromatin reader that primarily binds to H3K4me3-K9me3 (ref. ^6^). The prevailing assumption is that all the molecular events required for piRNA-directed DNA methylation occur after the engagement of MIWI2. We find that SPIN1 expression precedes that of both SPOCD1 and MIWI2. Furthermore, we demonstrate that young LINE1 copies, but not old ones, are marked by H3K4me3, H3K9me3 and SPIN1 before the initiation of piRNA-directed DNA methylation. We generated a *Spocd1* separation-of-function allele in the mouse that encodes a SPOCD1 variant that no longer interacts with SPIN1. We found that the interaction between SPOCD1 and SPIN1 is essential for spermatogenesis and piRNA-directed DNA methylation of young LINE1 elements. We propose that piRNA-directed LINE1 DNA methylation requires a developmentally timed two-factor authentication process. The first authentication is the recruitment of SPIN1–SPOCD1 to the young LINE1 promoter, and the second is MIWI2 engagement with the nascent transcript. In summary, independent authentication events underpin the precision of piRNA-directed LINE1 DNA methylation.

## Abstract (web summary)

In male mouse germline development, the precise DNA methylation of young, active transposons requires a two-step process in which SPIN1 and SPOCD1 mark young LINE1 elements before the piRNA pathway triggers DNA methylation.

## Main

Young active transposable elements (transposons) are a fundamental threat to the germline. The mouse genome is currently battling LINE1 and intracisternal A particle (IAP) elements^7–9^, and failure to silence transposons in the germline results in infertility^10–12^. DNA methylation is a key mechanism that represses transposons^13^. However, this presents a major vulnerability to the germline because DNA methylation is erased and reset during germ cell development^14^. The piRNA pathway defends the germline during this period of hypomethylation when transposons are expressed^1^ by post-transcriptionally silencing young active transposons and directing their DNA methylation^1^. piRNAs are small RNAs that are bound to PIWI proteins^1^. Through base complementarity, piRNAs guide the PIWI protein MILI to cleave transposon transcripts in the cytoplasm, neutralizing the immediate threat^2,15,16^. In the nucleus, piRNAs identify active transposons and tether MIWI2 to these nascent transcripts^2,3^. This sets in motion a series of events that culminate in the recruitment of the de novo methylation machinery. We previously defined MIWI2 interactomes from fetal gonocytes^3,4^ and found that SPOCD1 is an essential factor that connects the piRNA and de novo methylation machineries in vivo^4,5^. piRNA-directed transposon methylation requires precision. Failing to methylate every active transposon would be detrimental to the genomic integrity of the germline, but aberrant off-target methylation could result in germline-transmitted epimutations. piRNAs endow MIWI2 with the specificity to identify active transposon loci and, through tethering, trigger silencing. However, whether other mechanisms contribute to identifying active transposons and the exacting precision of the pathway remains unknown.

**Fig. 1:**
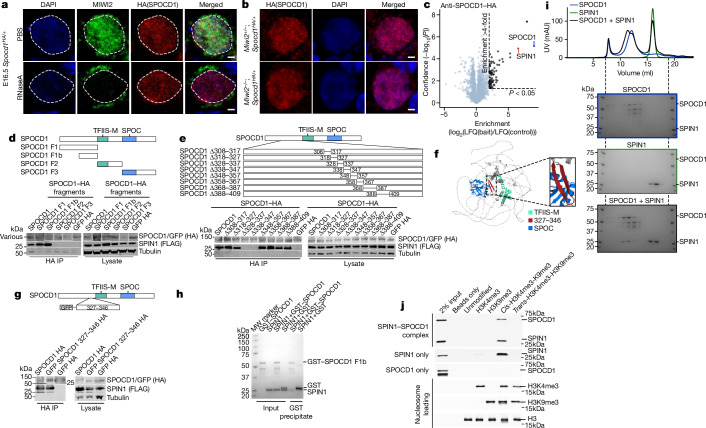
SPOCD1 directly interacts with the chromatin reader SPIN1. **a**, MIWI2 (green), haemagglutinin epitope tag (HA, red) and DAPI (blue) staining of E16.5 fetal testis sections from *Spocd1*^*HA*/*+*^ mice treated with PBS or RNase A before fixation. **b**, HA (red) and DAPI (blue) staining of E16.5 foetal testis sections from E16.5 *Miwi2*^−/−^*;Spocd1*^*HA*/+^ and *Miwi2*^+/−^*;Spocd1*^*HA*/+^ mice. Images in **a** and **b** are representative of *n* = 3 biological replicates; scale bars, 2 μm. **c**, Volcano plot showing enrichment (log_2_(mean label-free quantification ratio of anti-HA immunoprecipitates from *n* = 4 *Spocd1*^*HA*/*HA*^*/*wild-type) E16.5 fetal testes) and statistical confidence (−log_10_(*P*-value of two-sided Student’s *t*-test)) of proteins co-purifying with HA–SPOCD1 (data from ref. ^4^). **d**,**e**, Representative western blot analyses of *n* = 3 immunoprecipitations of the indicated SPOCD1 constructs with SPIN1 in HEK 293 T cells, for fragments (**d**) and specific deletions of amino acids (**e**). F, fragment. **f**, AlphaFold2 structure prediction of mouse SPOCD1 (B1ASB6) with key domains indicated. **g**, Representative western-blot analyses of *n* = 3 immunoprecipitations of the indicated mouse SPOCD1 constructs with SPIN1 from HEK 293 T cells. **h**, Representative Coomassie gel image of *n* = 3 co-precipitation experiments with the indicated recombinant proteins. **i**, Analytical size-exclusion chromatography of the SPOCD1–SPIN1 complex. Top, a representative chromatogram for each of the runs superposed. The Coomassie gels of each run are shown below. Samples from the same set of fractions were loaded on each gel (*n* = 2). Gel images to scale with chromatogram–elution volume corresponding to the outer lanes indicated by dashed lines. **j**, Nucleosome pull-down assays with site-specifically modified nucleosomes and recombinant SPIN1–SPOCD1 complex. Western blot images are representative of *n* = 3 independent pull-down experiments. For whole blot source data of **d**,**e**,**g**,**j** see Supplementary Fig. 1.

**Extended Data Fig. 1:**
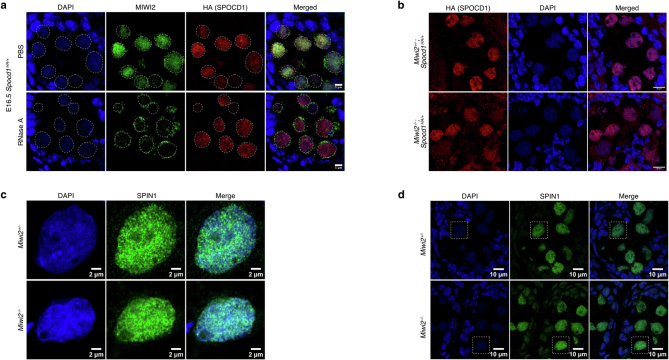
SPOCD1’s recruitment to chromatin is independent of MIWI2. **a**, MIWI2 (green), HA (red) and DAPI (blue) staining of E16.5 foetal testis sections from *Spocd1*^*HA/+*^ mice treated with PBS or RNase A prior to fixation. **b**, HA (red) and DAPI (blue) staining of E16.5 foetal testis sections from E16.5 *Miwi2*^*−/−*^*;Spocd1*^*HA/+*^ and *Miwi2*^*+/−*^*;Spocd1*^*HA/+*^ mice. **c**, **d**, SPIN1 (green) and DAPI (blue) staining of E16.5 *Miwi2*^*+/−*^ and *Miwi2*^*−/−*^ E16.5 foetal testis sections. (**c**) shows a zoom-in of the cell highlighted with a dashed rectangle in (**d**). Images of (**a**-**d**) are representative of *n* = 3 biological replicates. Scale bars are 5 μm (**a**), 10 μm (**b**, **d**) and 2 µm (**c**).

SPOCD1 accumulates in the nucleus before the expression of MIWI2 during male germ-cell development^4^. SPOCD1 expression is first observed in fetal gonocytes at embryonic day 14.5 (E14.5)^4^, whereas MIWI2 appears a day later and is mostly cytoplasmic until E16.5, when a considerable nuclear fraction is observed^4,15^. This pattern of successive accumulation could indicate that the recruitment of SPOCD1 to transposons may occur independently of MIWI2. Treating unfixed fetal gonocytes with RNase A results in the loss of nuclear MIWI2 staining^3^. Interestingly, the nuclear localization of SPOCD1 is insensitive to RNase A treatment (Fig. 1a and Extended Data Fig. 1a). Furthermore, MIWI2 deficiency does not affect SPOCD1 nuclear localization (Fig. 1b and Extended Data Fig. 1b). Together, these observations indicate that the recruitment of SPOCD1 to chromatin is independent of MIWI2. SPOCD1 does not contain any known chromatin-binding domains, so we examined the SPOCD1 immunoprecipitation mass spectrometry (IP-MS) data from E16.5 fetal testis for chromatin-binding proteins and found SPIN1 as a highly enriched, high-confidence associated protein^4^ (Fig. 1c). SPIN1 is a chromatin reader that comprises three Tudor-like domains (TLD1–3). It is a high-affinity H3K4me3 reader, and TLD2 recognizes this transcription-associated chromatin mark^17–19^. TLD1 binds heterochromatin-associated H3K9me3 (refs. ^20,21^), and this interaction increases the overall affinity of SPIN1 chromatin binding^6^. TLD3 does not contain an aromatic cage and mediates interactions with other proteins^6^. *Miwi2* deficiency does not affect SPIN1 nuclear localization in E16.5 fetal gonocytes (Extended Data Fig. 1c,d). We next sought to identify which portion of SPOCD1 is required to associate with SPIN1. To this end, we co-expressed SPOCD1, or fragments of it, with SPIN1 in HEK cells and tested their ability to interact. Full-length SPOCD1 and the amino-terminal 1–409 amino acids (fragment 1) co-precipitated SPIN1 (Fig. 1d). The interaction was further mapped to amino acids 205–409 (fragment 1b) (Fig. 1d). By sequentially deleting segments 10–20 amino acids long from the interacting fragment of SPOCD1, we mapped the SPIN1 association region to 20 amino acids (328–347) (Fig. 1e). These amino acids of SPOCD1 are predicted by the AlphaFold2 model to fold into a β-hairpin^22,23^ (Fig. 1f). Furthermore, fusion of 20 amino acids (327–346) with GFP revealed a SPIN1 interaction (Fig. 1g). We further demonstrated that the SPOCD1–SPIN1 interaction can be recapitulated using recombinant proteins. (Fig. 1h,i). Finally, using recombinant nucleosomes with distinct tail modifications in pull-down assays, we demonstrate that the SPOCD1–SPIN1 complexed protein fragments are pulled down only by *cis*-H3K4me3-K9me3 modifications (*cis* indicates that both modifications are on the same histone tail in the nucleosome) but not by H3K4me3 alone or by *trans*-H3K4me3-H3K9me3 (modifications on different histone tails) (Fig. 1j). In summary, SPOCD1 interacts with SPIN1, and the complexed proteins preferentially recognize the *cis*-H3K4me3-K9me3 chromatin mark.

**Fig. 2:**
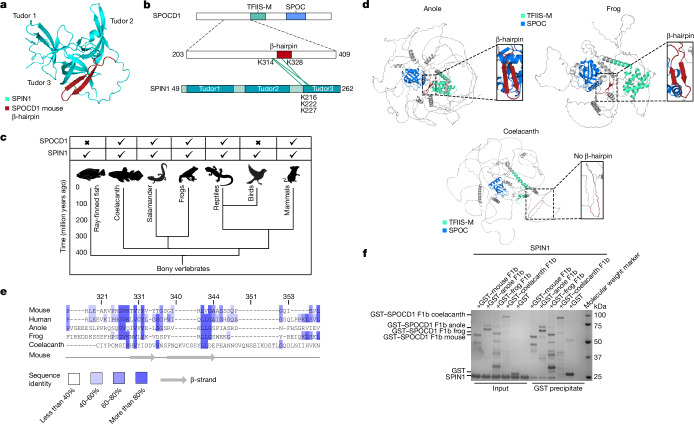
The SPOCD1–SPIN1 interaction is conserved. **a**, AlphaFold2 co-folding prediction of the interaction between SPIN1 (Q61142) and SPOCD1 (B1ASB6; only amino acids 326–348 are shown). **b**, Crosslinking mass spectrometry of mouse SPOCD1 fragment 1b (amino acids 203-409) with mouse SPIN1 (amino acids 49–262). Crosslinks are shown in green. **c**, Phylogenetic tree from ray-finned fishes to mammals showing the presence of SPOCD1 and SPIN1 in the indicated animal clades. **d**, AlphaFold2 prediction of SPOCD1 from *Anolis carolinensis* (an anole lizard, XP_008116112.1, amino acids 183–1397), *Xenopus tropicalis* (frog, XP_031752218.1) and *Latimeria chalumnae* (coelacanth, JH127468.5). The SPOC domain, TFIIS-M domain and SPIN1-interacting β-hairpin are highlighted. **e**, Multiple sequence alignment of the SPOCD1 SPIN1-interacting β-hairpin region from different species. Numbering for mouse SPOCD1 is shown above the sequences and secondary-structure elements of mouse SPOCD1 are shown below. Sequences are coloured according to sequence identity. **f**, Representative Coomassie gel image of *n* = 3 co-precipitation experiments with the indicated recombinant SPOCD1 from different species with mouse SPIN1.

**Extended Data Fig. 2:**
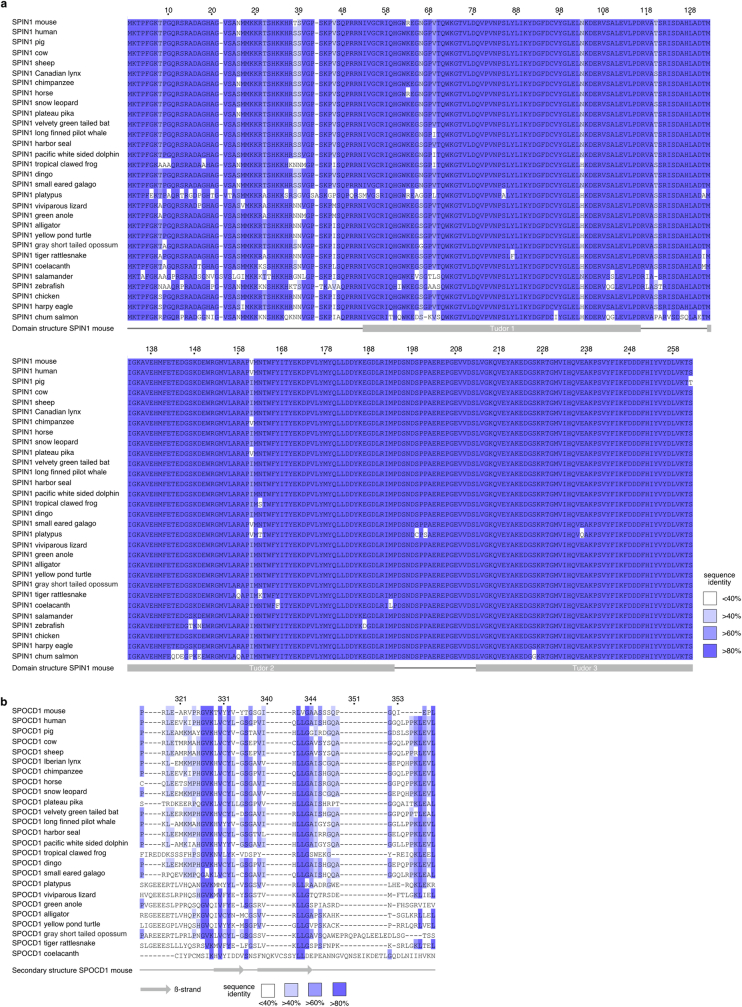
Multiple Sequence alignment of SPIN1 and the SPOCD1 ß-hairpin region. **a**, Multiple sequences alignment of SPIN1 from representative vertebrates. The domain structure of mouse SPIN1 (Q61142) is indicated underneath the alignment in grey. **b**, Multiple sequence alignment of the SPOCD1 ß-hairpin region with representative vertebrate SPOCD1 sequences. Secondary structure elements from the AlphaFold2 model of mouse SPOCD1 (B1ASB6) are shown below with grey arrows representing a ß-strand. **a**-**b**, sequences are coloured according to sequence identity. Numbering above according to mouse sequence.

Using AlphaFold2 (refs. ^22,23^) to model the co-folding of SPOCD1 and SPIN1 led to the prediction that the SPOCD1 β-hairpin interacts with the TLD3 of SPIN1 (Fig. 2a). Crosslinking mass spectrometry confirmed this prediction with crosslinks found adjacent to the β-hairpin of SPOCD1 and the TLD3 of SPIN1 (Fig. 2b). SPIN1 is a highly conserved protein (Extended Data Fig. 2a) and is found throughout vertebrates (Fig. 2c). We could retrieve full-length SPOCD1 coding sequences for the coelacanth (*Latimeria chalumnae*), the green anole lizard (*Anolis carolinensis*) and the tropical clawed frog (*Xenopus tropicalis*), but not from a salamander (*Axolotl mexicanum*). SPOCD1 apparently first arose in vertebrates, with orthologues found in the coelacanth but not in ray-finned fish, and it was later lost in birds^4^ (Fig. 2c). The SPOCD1 orthologues are predicted to have a similar overall fold to mouse SPOCD1, but only the lizard and the frog retain the conserved sequence and predicted β-hairpin structure that mediates the SPIN1 interaction in mice (Fig. 2d). Indeed, the sequence alignment revealed the coelacanth sequence to be less closely related to the other orthologues in this region (Fig. 2e and Extended Data Fig. 2b). Finally, we demonstrate that the frog and lizard SPOCD1–SPIN1 interaction can be reconstituted using recombinant proteins (Fig. 2f). In summary, SPOCD1 interacts with the chromatin reader SPIN1, and this association is conserved from amphibians to mammals.

**Fig. 3:**
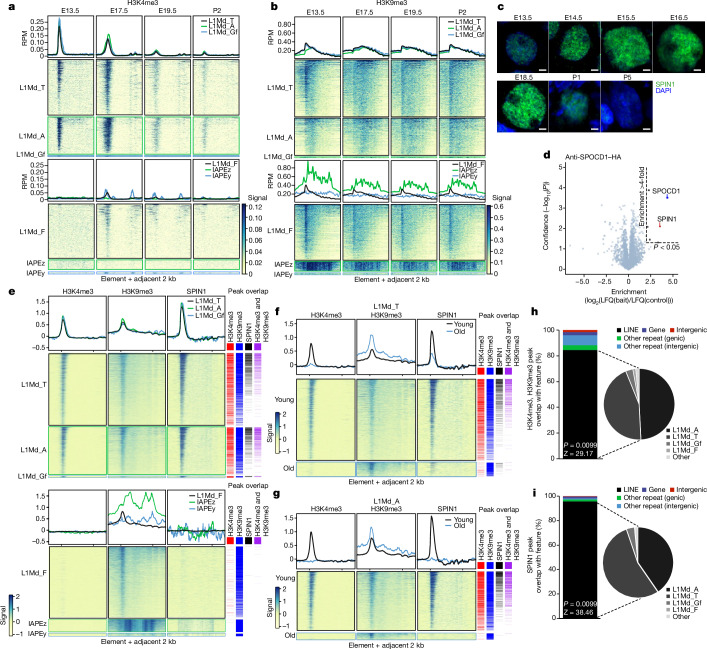
H3K4me3, H3K9me3 and SPIN1 mark young LINE1 elements before de novo genome methylation. **a**,**b**, Metaplot and heat map for different transposon families of H3K4me3 (**a**) and H3K9me3 (**b**) ChIP signal in reads per million (RPM) from fetal gonocytes at the indicated time points during mouse development. Data are merged from *n* = 2 biological replicates, reanalysed from ref. ^24^. **c**, SPIN1 (green) and DAPI (blue) staining of wild-type fetal testis sections from the indicated developmental time points. Images are representative of *n* = 3 biological replicates. Scale bars, 2 μm. **d**, Volcano plot showing enrichment (log_2_(mean label-free quantification ratio of anti-HA immunoprecipitates from *Spocd1*^*HA*/*HA*^*/*wild-type)) and statistical confidence (−log_10_(*P*-value of two-sided Student’s *t*-test)) of proteins co-purifying with HA-SPOCD1 from E14.5 fetal testes; *n* = 3. **e**–**i**, CUT&Tag data for H3K4me3, H3K9me3 and SPIN1 from E14.5 fetal germ cells. Data are merged from two (H3K4me3, H3K9me3) and three (SPIN1) biological replicates. In **e**–**g**, metaplot and heatmaps of signal over elements of different transposon families (**e**) are shown as well as young and old copies in the L1Md_T (**f**) and L1Md_A (**g**) families. Columns adjacent to the heatmaps show statistically significant peaks called for SPIN1 and the indicated histone modifications. In **e**, the overlap of H3K4me3 and H3K9me3 peaks with SPIN1 peaks is significant for L1Md_A (*P* = 0.0099, *Z*-score = 1,052), L1Md_T (*P* = 0.0099, *Z*-score = 1,398) and L1Md_Gf (*P* = 0.0099, *Z*-score = 2,007) by one-tailed permutation tests. In **f** and **g**, enrichment of overlapping H3K4me3 and H3K9me3 peaks with SPIN1 peaks is significantly different between young and old L1Md_A (adjusted *P* < 2.2 × 10^−16^) and L1Md_T (adjusted *P* < 2.2 × 10^−16^) copies, as observed by two-tailed Fisher’s exact test. In **h** and **i**, charts show overlap analysis of H3K4me3 and H3K9me3 peaks (**h**) and SPIN1 peaks (**i**) with the indicated genomic features. *P*-values and *Z*-scores from one-tailed permutation tests to assess the statistical significance of overlaps of CUT&TAG peaks with LINE1 elements are shown.

**Extended Data Fig. 3:**
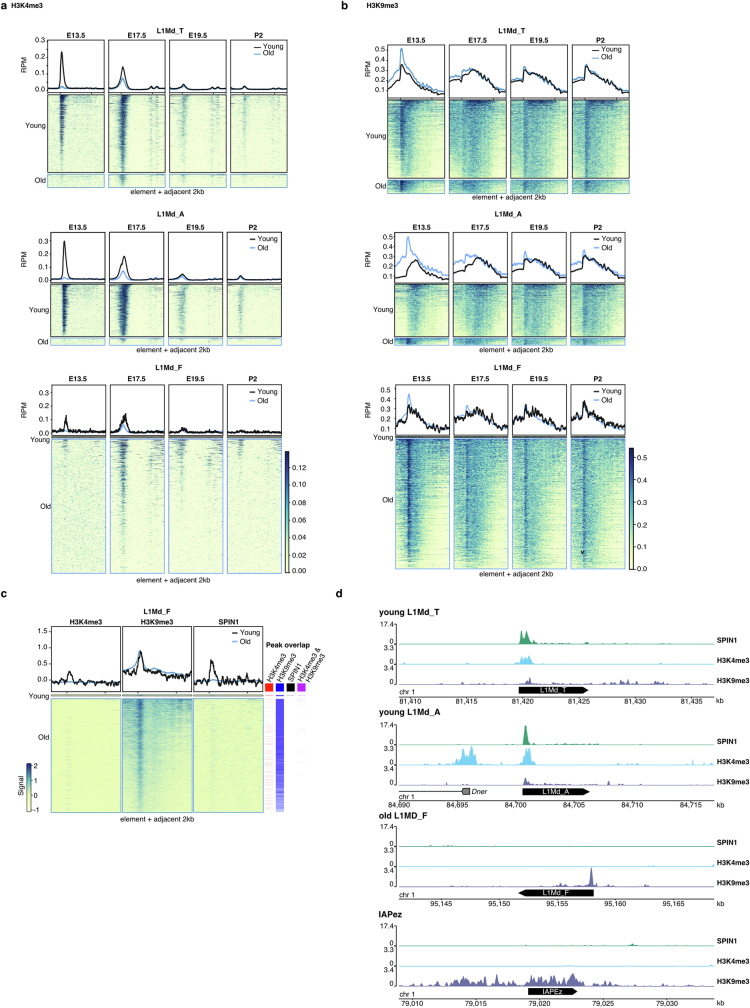
H3K4me3, H3K9me3 and SPIN1 mark young LINE1 elements prior to de novo genome methylation. Metaplot and heat maps of H3K4me3 (**a**) and H3K9me3 (**b**) ChIP from foetal gonocytes at the indicated timepoints during mouse development. Data is merged from two biological replicates, reanalysed from^24^. **a**-**b**, Panels show H3K4me3 (**a**) and H3K9me3 (**b**) ChIP-seq signal in reads per million (RPM) over young and old elements within the indicated LINE1 family. **c**, Metaplot and heat maps of indicated CUT&Tag signal of H3K4me3, H3K9me3 and SPIN1 over young and old L1MD_F elements. Columns adjacent to the heatmaps show peaks called for SPIN1 and the indicated histone modifications. Data is merged from two (H3K4me3, H3K9me3) and three (SPIN1) biological replicates. **a**-**c**, Data depicts element plus adjacent 2 kb for each of the transposon families indicated. **d**, Genome snapshots showing datatracks of CUT&Tag signal of H3K4me3, H3K9me3 and SPIN1 over selected genome regions containing a young L1Md_A, young L1Md_T, old L1Md_F or IAPEz element. Data is merged from two (H3K4me3, H3K9me3) and three (SPIN1) biological replicates. Enrichment of overlapping H3K4me3 and H3K9me3 peaks with SPIN1 peaks is not significantly different between young and old L1Md_F copies, as observed by a two-tailed Fisher’s exact test.

**Extended Data Fig. 4:**
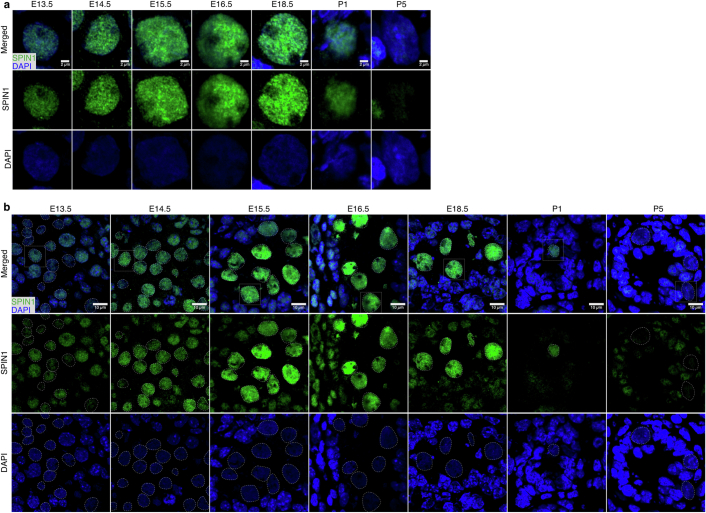
SPIN1 expression and localization in the developing mouse germline. **a**, **b**, Representative images of sections from *n* = 3 wild-type foetal testis stained for SPIN1 (green) and DAPI (blue) from indicated timepoints. Cell shown in (**a**) is highlighted with a white box in (**b**). Scale bars are 2 μm (**a**) and 10 μm (**b**).

We next sought to understand whether H3K4me3 and/or H3K9me3 mark young active transposons, which are the targets of the piRNA pathway. We reanalysed ChIP-seq data from fetal gonocytes purified from several developmental time points^24^. At E13.5, before the expression of the piRNA pathway and de novo methylation, the genome is fully demethylated. The process of genome and transposon methylation is occurring at E17.5, whereas by E19.5 and postnatal day 2 (P2), the bulk of genomic methylation has been completed^16,25^. We examined H3K4me3 for both young and old transposon families. We found that the young LINE1 families (L1Md_T, L1Md_Gf and L1Md_A), but not the old family L1Md_F, were enriched in H3K4me3 at E13.5, before the onset of de novo methylation (Fig. 3a). This enrichment was diminished but still present at E17.5, and was lost thereafter (Fig. 3a). H3K4me3 enrichment was not observed for the IAPEz and IAPEy families at E13.5 (Fig. 3a). Next, we analysed H3K9me3, for which the IAP families showed a high level of enrichment for all time points (Fig. 3b). Both young and old LINE1 families showed a peak of H3K9me3 across the promoter region at E13.5, and thereafter the enrichment extended across the body of the element (Fig. 3b). The young transposon families contain both young active elements and older inactive copies, which can be roughly distinguished by their divergence from their consensus sequence. We segregated young and old copies in LINE1 families and analysed H3K4me3 and H3K9me3 enrichment. Strikingly, H3K4me3 promoter enrichment is observed only in young LINE1 copies at E13.5 (Extended Data Fig. 3a). H3K9me3 enrichment was greater in older copies than in their younger counterparts at E13.5, after which old and young elements showed similar levels of enrichment (Extended Data Fig. 3b). In summary, young LINE1 elements are marked by both H3K4me3 and H3K9me3 before piRNA-directed DNA methylation. The prevailing view is that engagement of MIWI2 with the nascent transcript is the trigger for all downstream processes that culminate in DNA methylation. However, the fact that young LINE1 elements show a distinct chromatin modification pattern before de novo genome methylation challenges this view. We proposed that H3K4me3-K9me3 recruits SPIN1 and in turn SPOCD1 to young LINE1 elements before the engagement of MIWI2, and that this event licences the element for methylation. In support of this hypothesis, we found that SPIN1 is expressed in fetal gonocytes at E13.5 (the earliest time point analysed) and throughout the process of de novo genome methylation (Fig. 3c and Extended Data Fig. 4). Furthermore, we show that SPOCD1 associates with SPIN1 in E14.5 fetal gonads (Fig. 3d and Extended Data Table 1). We chose E14.5 for this experiment because it is the earliest time point at which SPOCD1 is expressed and is before the expression of MIWI2 and piRNA-directed DNA methylation^4^. We next optimized CUT&Tag^26^ for histone modifications and used SPIN1 from E14.5 fetal gonocytes. As we had observed at E13.5, H3K4me3 and H3K9me3 marked young LINE1 families and copies at this time point (Fig. 3e–g and Extended Data Fig. 3c,d). The CUT&Tag and ChIP-seq analyses cannot distinguish whether H3K4me3 and H3K9me3 mark a given locus in the same cell. Strikingly, SPIN1 was also found at young LINE1 families and copies (Fig. 3e–g and Extended Data Fig. 3c,d). The vast majority of sites in which H3K4me3 and H3K9me3 co-occur were LINE1 elements, predominantly from young families, followed by other repetitive elements and finally a handful of genes (Fig. 3h). SPIN1 occupancy showed a similar pattern of enrichment (Fig. 3j). In summary, we show that young LINE1 elements are enriched for H3K4me3, H3K9me3 and SPIN1 before the expression of MIWI2.

**Fig. 4:**
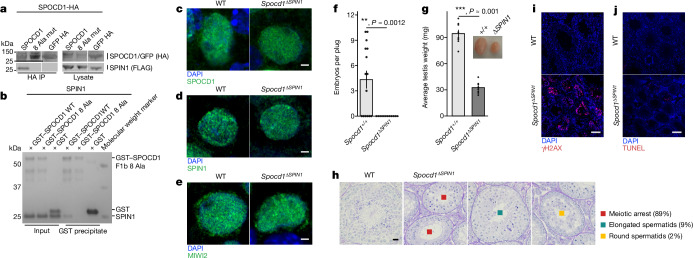
The SPOCD1–SPIN1 interaction is essential for spermatogenesis. **a**, Representative western-blot analyses of *n* = 3 immunoprecipitations of the mouse wild type and eight SPOCD1 alanine mutations (8 Ala mut) with SPIN1 in HEK 293 T cells. For whole-blot source data, see Supplementary Fig. 1. **b**, Representative Coomassie gel image of *n* = 3 co-precipitation experiments with the indicated recombinant proteins. **c**–**e**, Representative images of E16.5 gonocytes from *n* = 3 wild-type (WT) and *Spocd1*^*ΔSPIN1*^ mice stained for DNA (blue) and SPOCD1 (**c**), SPIN1 (**d**) or MIWI2 (**e**) (green). Scale bars, 2 μm. **f**, Number of embryos per plug fathered by studs with the indicated genotype mated to wild-type females. Data are mean and s.e.m. from *n* = 6 wild-type (15 plugs in total) and *n* = 6 *Spocd1*^*ΔSPIN1*^ studs (12 plugs). **g**, Testis weight of adult mice with the indicated genotype. Data are mean and s.e.m. from *n* = 8 wild-type and *n* = 8 *Spocd1*^*ΔSPIN1*^ mice. Inset, a representative image of testes from wild-type (left) and *Spocd1*^*ΔSPIN1*^ (right) mice. *P*-values in **f** and **g** were determined by unadjusted two-sided Student’s *t*-test. **h**, Representative images of PAS and haematoxylin-stained testes sections of wild-type and *n* = 5 *Spocd1*^*ΔSPIN1*^ adult mice, with different types of spermatogenic arrest observed in the tubules of the *Spocd1*^*ΔSPIN1*^ testes indicated. The percentage of each type of tubule is noted alongside. Scale bar, 20 μm. **i**,**j**, Adult testis sections stained for the DNA damage marker γH2AX (red) (**i**) and apoptotic cells (red) by TUNEL assay (**j**) from wild-type and *Spocd1*^*ΔSPIN1*^ mice (representative of *n* = 3 mice per genotype for γH2AX and *n* = 2 wild-type plus *n* = 3 *Spocd1*^*ΔSPIN1*^ mice for TUNEL). DNA was stained with DAPI (blue). Scale bars, 100 μm.

**Extended Data Fig. 5:**
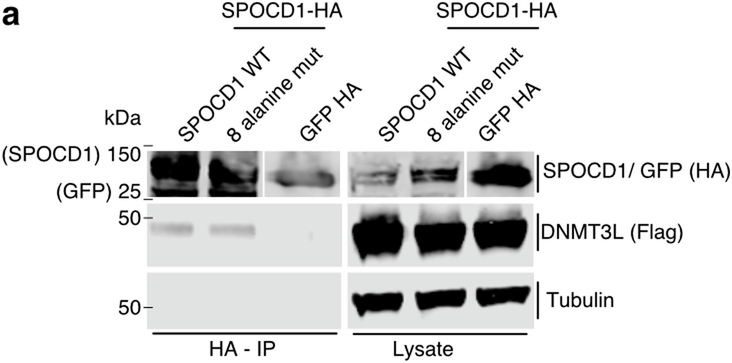
The SPOCD1-ΔSPIN1 separation-of-function protein associates with DNMT3L. **a**, Representative western blot analyses of *n* = 3 anti-HA immunoprecipitations of the HA epitope-tagged mouse wild-type, SPOCD1 8 alanine mutated proteins or GFP control with FLAG-tagged DNMT3L in HEK 293 T cells. For whole blot source data, see Supplementary Fig. 1.

**Extended Data Fig. 6:**
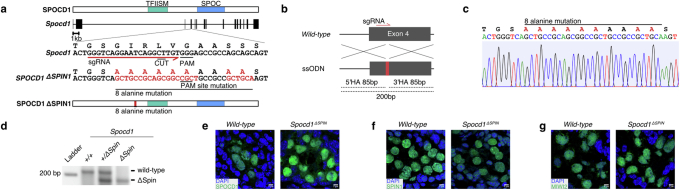
Generation of the *Spocd1*^*ΔSPIN1*^ mouse allele. **a**, Schematic representations of the mouse *Spocd1* locus and encoded 1015 amino acid protein are shown. sgRNA used for generation of the *Spocd1*^*ΔSPIN1*^ allele and adjacent PAM site are indicated. **b**, Schematic of CRISPR targeting strategy showing the location of single-stranded oligo DNA donor (ssODN) and homology arms (HA) used. **c**, Schematic representation, and sequencing trace of the part of *Spocd1*^*ΔSPIN1*^ exon 4 harbouring the mutation sites, a 30 bp sequence creating the 8 alanine mutation is highlighted in red. Sequencing was performed on *n* = 3 animals. **d**, Representative image of genotyping result for n = 3 *Spocd1*^*+/+*^, *Spocd1*^*+/ΔSPIN1*^ and *Spocd1*^*ΔSPIN1*^ mice. **e**-**g**, Representative images of E16.5 gonocytes from *n* = 3 *Spocd1*^*ΔSPIN1*^ and wild-type control mice stained for SPOCD1 (**e**), SPIN1 (**f**) or MIWI2 (**g**,) in green. DNA was stained with DAPI (blue). Scale bars are 5 μm.

SPIN1 is expressed beyond the germline, involved in other cellular processes, and required for mouse viability^27^. Therefore, we decided to identify a SPOCD1 separation-of-function mutation that uncouples SPOCD1 from SPIN1 to understand the importance of this interaction. Mutation of eight amino acids to alanine in one strand of the predicted β-hairpin that mediates SPIN1 binding abrogated the ability of SPOCD1 to co-precipitate SPIN1 when expressed in HEK cells (Fig. 4a). Furthermore, a recombinant SPOCD1 F1b fragment (Fig. 1d) with the 8 alanine mutation no longer interacts with recombinant SPIN1 (Fig. 4b). We termed this separation-of-function SPOCD1 mutant SPOCD1-ΔSPIN1. Importantly, like SPOCD1, the SPOCD1-ΔSPIN1 protein could associate with DNMT3L when both proteins are expressed in HEK cells (Extended Data Fig. 5a). We next engineered the *Spocd1*^*ΔSPIN1*^ mouse allele (Extended Data Fig. 6a–d). As is the case with *Spocd1*^*−/−*^ mice, *Spocd1*^*ΔSPIN1/ΔSPIN1*^ (termed *Spocd1*^*ΔSPIN1*^) mice are born in mendelian ratios from *Spocd1*^*ΔSPIN1/+*^ intercrosses and are indistinguishable from their wild-type litter mates (data not shown)^4^. The separation-of-function mutation did not affect the levels or localization of the SPOCD-ΔSPIN1 protein in *Spocd1*^*ΔSPIN1*^ E16.5 foetal testes compared to wild-type controls (Fig. 4c and Extended Data Fig. 6e). Furthermore, both SPIN1 as well as MIWI2 levels and localization in *Spocd1*^*ΔSPIN1*^ E16.5 foetal testes were indistinguishable from wild-type foetal testes (Fig. 4d,e and Extended Data Fig. 6f,g). In summary, the *Spocd1*^*ΔSPIN1*^ allele encodes a stably expressed SPOCD1 mutant protein and does not impact on SPIN1 or MIWI2 expression. *Spocd1*^*ΔSPIN1*^ male mice were infertile and have atrophic testes (Fig. 4f,g). Detailed histological analyses revealed a complex spermatogenic arrest (Fig. 4h). The vast majority of *Spocd1*^*ΔSPIN1*^ seminiferous tubules show a meiotic arrest that is typical of mutations that affect piRNA-directed transposon methylation (Fig. 4h). However, a small number of tubules show cells that have further developed to the round or elongated spermatid stage (Fig. 4h). The loss of the SPOCD1-SPIN1 interaction also results in DNA damage and apoptosis (Fig. 4i,j). In summary, the interaction of SPOCD1 with SPIN1 is essential for normal spermatogenesis and male fertility.

**Fig. 5:**
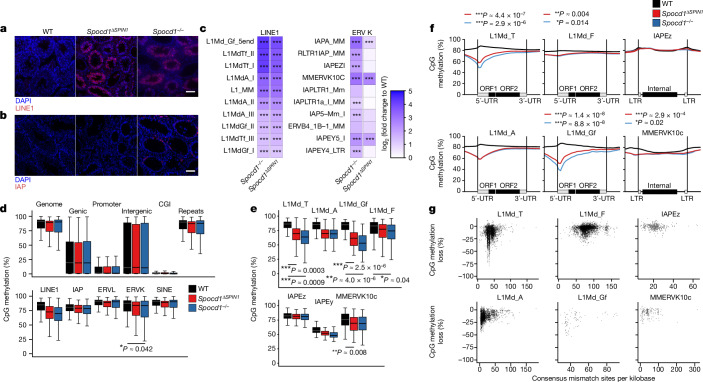
The SPOCD1–SPIN1 interaction is required for the de novo DNA methylation of young LINE1 elements. **a**,**b**, Representative testis sections of *n* = 3 wild-type, *Spocd1*^*ΔSPIN*^ and *Spocd1*^−/−^ mice stained red for the LINE1 ORF1p (**a**) or IAP GAG protein (**b**). DNA was stained with DAPI (blue). Scale bars, 100 μm. **c**, RNA-seq heat maps showing fold changes in expression relative to wild type for the ten most upregulated LINE1 and ERVK transposable elements in *Spocd1*^−/−^ P20 testes (*n* = 3 from each genotype). ***P < 0.001 of Bonferroni-corrected two-sided Wald’s test assuming n-binominal distribution. Only significant differences (*P* < 0.05) are shown. **d**–**g**, Genomic CpG methylation analysis of P14 undifferentiated spermatogonia from wild-type (*n* = 6), *Spocd1*^*ΔSPIN*^ (*n* = 4) and *Spocd1*^−/−^ mice (*n* = 3). **d**,**e**, Percentages of CpG methylation levels of the indicated genomic features (with genic, promoter and CpG island (CGI) regions defined as those not overlapping transposable elements, and intergenic regions as those not overlapping transposable elements or genes) or transposable elements (not overlapping genes) are shown as box plots. Boxes represent interquartile range from the 25th to the 75th percentile, the horizontal line shows the median, and whiskers show the data range of the median ± twice the interquartile range. Significant differences (*P* < 0.05 of Bonferroni-corrected two-tailed Student’s *t*-tests) of *Spocd1*^*ΔSPIN*^ (*n* = 4) and *Spocd1*^−/−^ (*n* = 3) samples to wild-type (*n* = 6) are indicated. **f**, Metaplots of mean CpG methylation over the indicated transposable element. *P = 0.05–0.01, **P = 0.01–0.001 and ***P < 0.001 for Bonferroni-corrected two-tailed Student’s *t*-tests comparing the average CpG methylation of the promoter region to wild type for *Spocd1*^*ΔSPIN1*^ (red) and *Spocd1*^−/−^ (blue). Only significant differences (*P* < 0.05) are shown. **g**, Correlation analysis of mean CpG methylation loss relative to the wild type for individual transposable elements of the indicated LINE1 and ERVK families in relation to their divergence from the consensus sequence in *Spocd1*^*ΔSPIN*^ spermatogonia.

The spermatogenic arrest in *Spocd1*^*ΔSPIN1*^ mice is indicative of defective transposon silencing and DNA methylation. In agreement with the selective marking of young LINE1 families with H3K4me3, H3K9me3 and SPIN1 before the expression of MIWI2, we found the expression of LINE1 ORF1p, but not IAP GAG, in *Spocd1*^*ΔSPIN1*^ adult testis (Fig. 5a,b). Furthermore, RNA sequencing (RNA-seq) from P20 testis confirmed that the same LINE1 families are deregulated in *Spocd1*^*ΔSPIN1*^ and *Spocd1*^−/−^ mice (Fig. 5c). This analysis also confirmed the lack of deregulated expression of evolutionarily young IAP families in *Spocd1*^*ΔSPIN1*^ mice (Fig. 5c). We next analysed genome methylation from purified P14 spermatogonia, a time point used in previous analyses^3,4^ because it is before the onset of spermatogenic arrest but after the completion of de novo genome methylation. The piRNA pathway and SPOCD1 are specifically required for the de novo DNA methylation of young LINE1 and IAP elements^3,4,10,12,28–30^. Accordingly, genome de novo methylation is normal in *Spocd1*^*ΔSPIN1*^ adult testis (Fig. 5d). Indeed, the loss of the SPOCD1–SPIN1 interaction did not affect genic, intergenic, CpG island and gene-promoter regions, or collective transposon DNA methylation levels (Fig. 5d). The young LINE1 families L1Md_A, L1Md_Gf and L1Md_T were not fully methylated in *Spocd1*^*ΔSPIN1*^ spermatogonia, whereas almost normal levels of methylation were observed for the young IAPEz family (Fig. 5e). The piRNA pathway directs DNA methylation at the promoters of transposons^28^. A metaplot analysis of methylation levels from *Spocd1*^*ΔSPIN1*^ spermatogonia revealed defective de novo promoter methylation specifically in young LINE1 families such as L1Md_T, L1Md_A and L1Md_Gf compared with the older L1Md_F family and the IAPEz family (Fig. 5f). The overall reduction in promoter methylation in *Spocd1*^*ΔSPIN1*^ cells is similar to that observed in *Spocd1*^−/−^ mice^4^ (Fig. 5f). We next looked at the loss of methylation at individual transposon loci as a function of their divergence from the consensus sequence, which is a proxy for age. This analysis confirmed that the SPOCD1–SPIN1 interaction is required for the methylation of young LINE1 elements in the respective families (Fig. 5g). IAPEz element methylation was unaffected in *Spocd1*^*ΔSPIN1*^ spermatogonia (Fig. 5g). In summary, the SPOCD1–SPIN1 interaction is predominantly required for the piRNA-directed DNA methylation of young LINE1 elements.

Here we show that SPOCD1 interacts directly with the chromatin reader SPIN1 and that this interaction arose early in tetrapod evolution. H3K4me3, which is the key determinant of SPIN1 chromatin association^17,18^, specifically marks young LINE1 elements. H3K9me3, which biochemically augments SPIN1 chromatin binding^6^, is found at the same elements. H3K4me3 is associated with transcription^19^ so the presence of this modification at young LINE1s is due to their expression. However, the mechanism by which H3K9me3 is deposited remains undetermined. We demonstrate that SPOCD1–SPIN1 complexed protein fragments have a higher affinity for *cis*-H3K4me3-K9me3 than for *trans*-H3K4me3-H3K9me3 or H3K4me3-modified recombinant nucleosomes. We also found that the co-occurrence of both H3K4me3 and H3K9me3 is predominantly a transposon-related chromatin feature in fetal gonocytes. Accordingly, we observed that most of the SPIN1 was bound to LINE1s. The recruitment of SPIN1 to LINE1s and the SPOCD1–SPIN1 interaction occur before the nuclear localization of MIWI2 and the process of de novo DNA methylation. We demonstrate that this interaction is required for spermatogenesis and piRNA-directed LINE1 methylation. The spermatogenic phenotype in *Spocd1*^*ΔSPIN1*^ mice differs from a deficiency of *Spocd1* or *Miwi2*, for which strict meiotic arrest is observed^4,12^. The basis of this difference could lie in the fact that only LINE1s are deregulated in *Spocd1*^*ΔSPIN1*^ mice, whereas defective LINE1 and IAP silencing are observed in *Spocd1*^−/−^ and *Miwi2*^−/−^ mice^4,12,30^. Interestingly, in mice for which the PIWI protein MILI has lost its endonuclease activity, a similar spermatogenic arrest is observed and only LINE1s are deregulated^2^. How SPOCD1 is recruited to IAPs remains unknown, but we speculate that another SPOCD1-associated protein could mediate this recruitment through the recognition of a distinct chromatin signature or sequence motif. The different mechanisms in LINE1 and IAPs reveals an unexpected complexity in the pathway. The prevailing notion is that all the molecular events required for piRNA-directed DNA methylation occur after the engagement of the piRNA–MIWI2 ribonucleoprotein complex with the nascent transcript. Here, we demonstrate that multiple independent and developmentally choreographed events are required for LINE1 piRNA-directed DNA methylation. Our revised model posits that the recruitment of SPIN1–SPOCD1 through chromatin modification to young LINE1 elements constitutes a first licensing step. The engagement of MIWI2 with the nascent transcript is the second licensing event and triggers DNA methylation. In summary, we propose that a two-factor authentication system ensures the precision of LINE1 piRNA-directed methylation.

## Methods

### Mouse strains and experimentation

The *Spocd1*^*HA*^ and *Miwi2*^*tdTomato*^ (*Miwi2*^*tdTom*^) mouse alleles have been described previously^4,31^. *Miwi2*^*tdTom*^ is a *Miwi2* null allele and is used as such^31^. Both lines were kept on a mixed B6CBAF1/Crl;C57BL/6 N;Hsd:ICR (CD1) genetic background. The *Spocd1*^*ΔSPIN1*^ allele was generated by CRISPR–Cas9 gene editing as previously described^32,33^. A single guide RNA (sgRNA) (GGGTCAGGAATCAGGCTTGT) together with Cas9 mRNA and a single-stranded DNA oligonucleotide containing the eight-alanine mutation flanked by 85 base pairs (bp) of homology arm (AGATGGTAAACAGTTGAAGCCAAGGCAGGGAGGATTTCAGGCAGAGCCTTGCCATACTCTCTCTCAGCAGGTCTACACTGGGTCAGCTGCCGCAGCGGCCGCTGCCGCCGCTGCAAGTCAGCCAGGACAAATTGAACCTCTGGAGGAGTTGGACACCAACTCAGCCAGAAGGAAGAGAAGGCCCACAACTGCTCACCCTA) was injected into the cytoplasm of fertilized single-cell zygotes (B6CBA F1/Crl). F_0_ offspring were screened by PCR and the *Spocd1*^*ΔSPIN1*^ allele was confirmed by Sanger sequencing. The allele was established from one founder animal and back-crossed several times to a C57BL/6N genetic background. The *Spocd1*^*ΔSPIN1*^ mice were thus on a mixed B6CBAF1/Crl;C57BL/6N genetic background. Animals were genotyped using a PCR of four primers (F, GACCCTGTATTTATTGAAGTCACTG; R, CCTCAGTGACATCAGGCGGA; WT-F, CACTGGGTCAGGAATCAGGC; and ∆Spin-R, GTCCTGGCTGACTTGCAGC). Mice carrying the *Oct4*^*eGFP*^ reporter allele^34^ were originally obtained from Jackson Laboratories (B6;129S4-Pou5f1^tm2Jae^/J (Oct4-eGFP), stock number 008214).

Male fertility was assessed by mating studs to Hsd:ICR (CD1) wild-type females and counting the number of pups born for each plugged female. For each experiment, animal tissue samples were collected from one or more litters and allocated to groups according to genotype. No further randomization or blinding was applied during data acquisition and analysis.

Animals were maintained at the University of Edinburgh, UK, in accordance with the regulation of the UK Home Office, or at the Institute for Molecular Biology in Mainz, Germany, in accordance with local and European animal-welfare laws. Ethical approval for the UK mouse experimentation has been given by the University of Edinburgh’s Animal Welfare and Ethical Review Body and the work done under licence from the UK Home Office. Animal experiments done in Germany were approved by the ethical committees on animal care and use of the federal states of Rheinland-Pfalz, Germany, covered by LUA licence G 23-5-049.

### Immunofluorescence

Immunofluorescence experiments were done as previously described^35^. The following primary antibodies were used in this study: anti-HA (Cell Signaling Technologies) 1:200; anti-LINE1-ORF1p (ref. ^36^) 1:500; anti-IAP-GAG (a gift from B. Cullen, Duke University) 1:500; anti-γH2AX (Bethyl Laboratories) 1:500; anti-MIWI2 (a gift from R. Pillai, Université de Genève) 1:500; anti-SPOCD1 rabbit serum rb175 1:500 (O’Carroll laboratory antibody); anti-SPIN1 (Cell Signaling Technologies) 1:500 (of a custom preparation of 1.1 μg μl^−1^ in PBS). Images were taken on a Zeiss Observer or Zeiss LSM880 with an Airyscan module. Images acquired using the Airyscan module were deconvoluted with the Zeiss Zen software ‘Airyscan processing’ with settings 3D and a strength of 6. ImageJ and Zeiss Zen software were used to process and analyse the images.

### Cell culture, transfection, immunoprecipitation and western blotting

HEK293T cells (O’Carroll laboratory stock, not further authenticated, tested for mycoplasma contamination) were cultured and transfected as previously described^4^ with a minor modification, and 3 μl Jetprime reagent was used. On day 2 after transfection, cells were washed twice with PBS and resuspended in 1 ml lysis buffer (IP buffer: 150 mM KCl, 2.5 mM MgCl_2_, 0.5% Triton X-100, 50 mM Tris-HCl, pH 8, supplemented with 1× protease inhibitors (cOmplete ULTRA EDTA-free, Roche) with 37 units per ml benzonase (Millipore)) and lysed for 30 min, rotating at 4 °C. The lysate was cleared by centrifugation for 10 min at 21,000*g*. Cleared lysate (800 μl) was incubated with 20 μl of anti-HA beads (Pierce) that had been calibrated in lysis buffer and incubated for 1 h at 4 °C on a rotating wheel. The beads were washed four times with lysis buffer. Immunoprecipitates were eluted at 50 °C for 10 min in 20 μl 0.1% sodium dodecyl sulphate (SDS), 50 mM Tris-HCl, pH 8. Lysates and eluates were run on a 4–12% bis–tris acrylamide gel (Invitrogen) and blotted onto a nitrocellulose membrane (Amersham Protran 0.45 NC) according to standard laboratory procedures. The membrane was blocked with blocking buffer (4% (w/v) skimmed milk powder (Sigma-Aldrich) in PBS-T (phosphate buffered saline, 0.1% Tween-20)) and subsequently incubated for 1 h with primary antibodies (anti-HA (C29F4s, Cell Signaling Technologies), 1:1,000; anti-FLAG (M2, Sigma-Aldrich) 1:1,000, anti-SPOCD1 rabbit serum rb175 (O’Carroll laboratory antibody) 1:500 or anti-α-Tubulin (T9026, Sigma-Aldrich) 1:1,000) in blocking buffer. The anti-α-tubulin staining was used as loading control on the same blot as the experimental staining. After three PBS-T washes for 10 min, the membrane was incubated with secondary antibodies (IRDye 680RD donkey anti-rabbit or IRDye 800CW donkey anti-mouse, LI-COR, 1:10,000) in blocking buffer for 1 h. It was washed three times for 10 min in PBS-T and imaged on a LI-COR Odyssey CLx system. Exposure of the entire images was optimized in Image Studio Lite (LI-COR), and areas of interest were cropped for presentation.

### Protein alignments and structure prediction

The mouse SPOCD1 AlphaFold2 protein structure prediction model^22,23^ was downloaded from the AlphaFold Protein Structure Database (https://www.alphafold.ebi.ac.uk/). Models for the SPOCD1–SPIN1 interaction, as well as the single SPOCD1 proteins from *Anolis*, *Xenopus* and *Latimeria*, were generated with AlphaFold2 (refs. ^22,23^) on ColabFold^37^. The model was visualized using PyMol^38^. Multiple sequence alignments of SPOCD1 and SPIN1 were generated with ClustalW^39^ and edited in Jalview^40^. For SPOCD1, alignments were edited based on secondary-structure elements of the AlphaFold2 model (B1ASB6) using Jalview^40^.

### Protein purification

GST-tagged mouse SPOCD1 fragments (amino acids 203–409), *Anolis* SPOCD1 fragments (XP_008116112.1, amino acids 457–748), *Xenopus* SPOCD1 fragments (XP_031752218.1, amino acids 1–229), *Latimeria* SPOCD1 fragments (XP_014348336.1, amino acids 510–1009) and His-tagged SPIN1 (amino acids 49–262) were cloned in a pET-based backbone. Proteins were expressed in *Escherichia coli* BL21 (DE3). Bacteria were grown in 2xTY media at 37 °C until an optical density of 0.8 was reached. Then, the temperature was reduced to 18 °C, the bacteria were induced with 1 mM IPTG and grown for another 14–16 h. Cells were collected and pellets were stored at −80 °C until purification. The pellets were resuspended in 50 ml lysis buffer (20 mM Tris-HCl, pH 7.5, 200 mM NaCl, 2.5 mM imidazole, 0.5 mM β-mercaptoethanol, Roche cOmplete EDTA-free Protease Inhibitor Cocktail, 0.01 mg ml^−1^ DNaseI (Sigma) and 2 mM AEBSF (Pefabloc) for SPIN1, or 20 mM Tris-HCl, pH 7.5, 200 mM NaCl, 1 mM DTT, Roche cOmplete EDTA-free Protease Inhibitor Cocktail, 0.01 mg ml^−1^ DNaseI (Sigma) and 2 mM AEBSF (Pefabloc) for SPOCD1) and cells were lysed with the Constant systems 1.1 kW TS cell disruptor at 25 kPSI. The cleared lysate was used to load on a cOmplete His-Tag Purification Column (Roche) for SPIN1 or incubated with 7 ml glutathione sepharose high-performance beads (Cytiva) for SPOCD1 calibrated in the respective buffer. Elution from column/beads with increasing (2.5–500 mM) imidazole gradient for SPIN1 or GST elution buffer containing 20 mM reduced glutathione for SPOCD1. The fractions of interest were pooled and dialysed overnight in 20 mM Tris-HCl, pH 7.5, 100–150 mM NaCl, 1 mM DTT. The SPIN1 construct was cleaved with GST–3C protease (made in our lab) overnight. The SPOCD1 constructs were concentrated and stored at −80 °C until used. SPIN1 was further purified by ion exchange with a gradient of 100–1,000 mM NaCl (Resource Q, Cytiva) and size-exclusion chromatography (HiLoad 16/600 Superdex 200 pg, Cytiva). Finally, the protein was concentrated and stored at −80 °C until used.

### Nucleosome pull-downs with recombinant SPIN1-SPOCD1 proteins

Histone H3 site-specifically modified with H3K4me3 and/or H3K9me3 was generated by native chemical ligation (NCL) and assembled into nucleosomes as described previously^41,42^. In brief, *Xenopus* H3 and H4 and human H2A and H2B were expressed in *E. coli* and purified from inclusion bodies. For NCL, a tail-less histone H3 lacking residues 1–31 and containing a threonine-to-cysteine substitution at position 32 and a cysteine-to-alanine substitution at position 110 of *Xenopus* H3 (H3Δ1–31T32C C110A) was expressed in *E. coli* and purified in the same way. NCL reactions were carried out with synthetic carboxy-terminal benzyl thioester peptides spanning residues 1–31 of histone H3.1 and carrying the desired modifications at K4 and K9 (Peptide Protein Research) in 6 M guanidine HCl, 250 mM sodium phosphate buffer, pH 7.2, 150 mM 4-mercaptophenylacetic acid (MPAA, Sigma) and 50 mM TCEP for 72 h at room temperature. Ligated full-length modified histone H3 was purified through cation-exchange chromatography on a HiTrap SP column (Cytiva). Histone octamers were reconstituted by dialysis and purified by gel filtration on an S200 size-exclusion column (Cytiva). For the generation of *trans*-histone octamers carrying H3K4me3 and H3K9me3 on separate copies of histone H3, the H3X–H3Y system was used^43^, starting from H3Δ1–31T32C C110A constructs that also contained the required H3X and H3Y mutations. H3X was used for H3K4me3 and H3Y for H3K9me3. A biotinylated 209-bp DNA fragment containing the 601 nucleosome positioning sequence was generated by PCR and purified by ion-exchange chromatography on a HiTrap Q column followed by ethanol precipitation. Mononucleosomes were then assembled from histone octamers and 601 DNA by gradient dialysis. Nucleosome assembly was verified by native gel electrophoresis on 6% acrylamide gels in 0.5× TGE buffer (12.5 mM Tris, pH 8.0, 95 mM glycine and 0.5 mM EDTA).

Nucleosome pull-down assays were done essentially as described previously^44^. All incubations and washes were performed at 4 °C with end-over-end rotation, and all centrifugation steps were done at 1,500*g* for 2 min at 4 °C. Then, 23 pmol (3 µg) of recombinant, site-specifically modified nucleosomes were bound to streptavidin sepharose high-performance beads (Cytiva) by overnight incubation in pull-down buffer (20 mM HEPES, pH 7.9, 175 mM NaCl, 10% glycerol, 1 mM EDTA, 1 mM DTT, 0.1% NP-40, 0.1 mg ml^−1^ BSA). Before incubation, beads were blocked with 1 mg ml^−1^ BSA in pull-down buffer. Nucleosome-bound beads were washed three times with pull-down buffer before incubation with recombinant SPIN1 and SPOCD1 proteins for 2 h. His-tagged SPIN1 (49–262) and His-tagged SPOCD1 fragment 1b were expressed and purified as above. SPIN1–SPOCD1 fragment 1b complexes were purified by size-exclusion chromatography on an S200 increase column (Cytiva) as above. For the experiment shown in Fig. 1j, 23 pmol of protein was used. After incubation with recombinant proteins, beads were washed three times with high-salt pull-down buffer (as above but with 350 mM NaCl) for 5 min. Nucleosomes and bound proteins were eluted by boiling in 1.5× SDS sample buffer (95 mM Tris HCl, pH 6.8, 15% glycerol, 3% SDS, 75 mM DTT, 0.15% bromophenol blue). Binding was analysed by western blotting with antibodies against His tag (Sigma H1029, lot 033m4785) 1:1,000. Antibodies against histone H3 (Abcam ab176842, lot GR1494741-36) 1:2,500, H3K4me3 (Cell Signaling) 1:2,000 and H3K9me3 (Abcam ab176916) 1:1,000 were used to verify nucleosome loading and modification state.

### Analytical size-exclusion chromatography

For analytical size-exclusion chromatography, 125 μg SPIN1 and/or 500 μg mouse GST–SPOCD1-F1b were used for each run. Proteins were diluted in 250 μl size-exclusion chromatography buffer (20 mM HEPES, pH 7.5, 150 mM NaCl, 1 mM DTT) and injected on a Superdex 200 10/300 GL column. Peak fractions were collected, loaded on an SDS–PAGE gel and visualized by Coomassie staining.

### Crosslinking mass-spectrometry analysis

Recombinant fragments (25 μg) of SPOCD1 (GST–F1b) and SPIN1 were incubated in 20 mM HEPES, pH 7.5, 150 mM NaCl, 1 mM DTT and crosslinked with BS3 (bis(sulfosuccinimidyl)suberate) (Thermo Fisher Scientific) at BS3:protein ratios of 1:1, 2:1 and 4:1 (w/w) for 2 h on ice. The crosslinking reaction was stopped by adding 2 μl ammonium bicarbonate (2.0 M). Crosslinking products were run on 4–12% bis-Tris NuPAGE (Invitrogen) for 15 min and briefly stained using Instant Blue (Expedeon). Bands at more than 150 kD were excised and the proteins were reduced with 10 mM DTT for 30 min at room temperature, alkylated with 55 mM iodoacetamide for 20 min at room temperature and digested using 13 ng μl^−1^ trypsin (Promega) overnight at 37 °C^37^. The digested peptides were loaded onto C18-Stage-tips^38^ for liquid chromatography with tandem mass spectrometry (LC-MS/MS) analysis. The LC-MS/MS analysis was performed using Orbitrap Fusion Lumos (Thermo Fisher Scientific) with a ‘high/high’ acquisition strategy. The peptide separation was done on an EASY-Spray column (50 cm × 75 μm internal diameter, PepMap C18, 2-μm particles, 100 Å pore size; Thermo Fisher Scientific). Mobile phase A consisted of water and 0.1% (v/v) formic acid. Mobile phase B consisted of 80% (v/v) acetonitrile and 0.1% (v/v) formic acid. Peptides were loaded at a flow rate of 0.3 μl min^−1^ and eluted at 0.25 μl min^−1^ using a linear gradient going from 2% mobile phase B to 40% mobile phase B over 102 or 132 min (each sample was run twice with different gradients), followed by a linear increase from 40% to 95% mobile phase B in 11 min. The eluted peptides were introduced directly into the mass spectrometer. MS data were acquired in the data-dependent mode with a 3 s acquisition cycle. Precursor spectra were recorded in the Orbitrap with a resolution of 120,000 and a mass-to-charge ratio (*m/z*) range of 350–1,700. Ions with a precursor charge state between 3+ and 8+ were isolated with a window size of *m*/*z* = 1.6 and fragmented using high-energy collision dissociation with a collision energy of 30. The fragmentation spectra were recorded in the Orbitrap with a resolution of 15,000. Dynamic exclusion was enabled with single repeat count and 60 s exclusion duration. The mass-spectrometric raw files were processed into peak lists using ProteoWizard (v.3.0)^39^ and crosslinked peptides were matched to spectra using Xi software (v.1.7.6.4)^40^ with in-search assignment of mono-isotopic peaks^41^. Search parameters were: MS accuracy, 3 ppm; MS/MS accuracy, 5 ppm; enzyme, trypsin; crosslinker, BS3; maximum missed cleavages, 4; fixed modification, carbamidomethylation on cysteine; variable modifications, oxidation on methionine; fragments b and y ions with loss of H_2_O, NH_3_ and CH_3_SOH. The linkage specificity for BS3 was assumed to be at lysine, serine, threonine, tyrosine and protein N termini. Identified candidates of crosslinked peptides were validated by Xi software^40^, and only auto-validated crosslinked peptides were used. Identified crosslinks underlying Fig. 2b are shown in Supplementary Table 1.

### ChIP sequencing analysis

Raw fastq.gz sequencing files for ChIP-seq of H3K4me3 and H4K9me3 were downloaded from the Sequence Read Archive record SRP165187 (ref. ^24^). Paired-end reads were preprocessed to remove adapter sequences and trim low-quality bases using Trimmomatic v.0.35 (ref. ^45^). Tru-seq adapter sequences were used in the case of ChIP-seq samples. Trimmed reads were aligned to the mouse mm10 genome with bwa mem v.0.7.16 (ref. ^46^) using the -M parameter. Alignments were filtered to remove duplicate reads with Picard MarkDuplicates v.2.24.0 (http://broadinstitute.github.io/picard/) and improper alignments with Samtools view v.1.11 -F 260 -f 3 (ref. ^47^). In the case of multi-mapping reads, a single alignment (marked as primary by bwa) was selected for downstream analysis. BAM files were converted to normalized bigWig files for visualization and plotting using deepTools^48^ bamCoverage v.3.5.0 with the following parameters: -bs 1 --normalizeUsing BPM.

### ChIP heatmaps and average profile plots

Genomic annotations for repetitive elements L1Md_A, L1Md_T, L1Md_F (combining elements classified as L1MD_F, L1Md_F2, L1Md_F3), L1Md_Gf, IAPEy and MMERVK_10C were extracted from Repeat Masker using the UCSC table browser. Normalized read coverage was computed across these elements using deepTools v.3.5.0 computeMatrix. The central regions were length-normalized to 5 kb with flanking regions ±2 kb from the start and end positions. Heatmaps were drawn using deepTools v.3.5.0 plotHeatmap, separating each repetitive element and sorting rows in descending order of total signal. LINE1 elements (L1Md_A, L1Md_F and L1Md_T) were further separated into young LINE1 elements based on a divergence of 38 bases per kb or less from a consensus sequence^4^ or the presence of an intact functional promoter denoted by the presence of specific monomer annotations^49^. Monomers associated with inert promoters (subtypes 6 and 2) were removed from the analysis. Average profiles were generated for each experiment and each category of repetitive element by calculating the mean signal between replicate samples. Computations were performed in R, with the seqplots package^50^, using bins of 50 bases, flanking regions of 2 kb and a central-region length normalized to 5 kb. Final plots were drawn and formatted using the tidyverse packages^51^.

### IP-MS

IP-MS of SPOCD1–HA from *Spocd1*^*HA*/+^ E14.5 fetal testis using 50 μl of anti-HA beads (Pierce, 88837) was done as previously described^4^, with a reduced number of 25 testes per replicate. Wild-type fetal testes were used as controls.

### Fluorescence-activated cell sorting (FACS)

To purify foetal germ cells for CUT&Tag analysis, E14.5 testes were dissected from embryos carrying the *Oct4*^*eGFP*^ allele^34^. A single cell suspension was obtained by sequential treatment with 100 µl collagenase solution at 37 °C for 8 min (10 units of collagenase A (Sigma-Aldrich 10103578001); 2× NEAAs (Gibco); 2× Na-pyruvate (Gibco); 25 mM HEPES–KOH, pH 7.5) and 200 µl TryPLE Express (Gibco) at 37 °C for 5 min with gentle flicking and pipetting of the solution to aid dissociation. Digestion was neutralized by 70 µl prewarmed FBS and cells were collected by spinning at 600*g* for 4 min at room temperature followed by two washes in FACS buffer (1× PBS; 2 mM EDTA, 25 mM HEPES-KOH, pH 7.5, 1.5% BSA, 10% FBS; 2 µg ml^−1^ DAPI) and filtering (Corning, 352235) just before sorting. Cell sorting was done on an Invitrogen Bigfoot using a 100 μm nozzle and gating for DAPI-negative (live), OCT4–eGFP-positive (germ cells) populations into collection tubes containing 100 µl 1× PBS.

For EM-seq, CD9^+^ spermatogonia were sorted from P14 testes as described previously^52^ using Fc block (eBioscience, 14-0161-86, clone 93, lot 2297433) 1:50; biotin-conjugated anti-CD45 (eBioscience, 13-0451-85, clone 30-F11, lot 2349865) 1:400, and biotin-conjugated anti-CD51 (Biolegend, 104104, clone RMV-7, lot B308465) 1:100 anti-CD9^APC^ (eBioscience, 17-0091-82, clone eBioKMC8, lot 2450733) 1:200, anti-cKit^PE-Cy7^ (eBioscience, 25-1171-82, clone 2B8, lot 2191977) 1:1,600, streptavidin^V450^ (BD bioscience, 560797, lot 1354158) 1:400 and 1 μg ml^−1^ DAPI. Cells were sorted into DMEM media on a BD Aria II sorter, pelleted for 5 min at 500*g* and snap frozen in liquid nitrogen.

For gating strategies, see Supplemental Fig. 2.

### CUT&Tag assays

CUT&Tag was done on FACS-isolated fetal germ cells as previously described^26^, with some minor modifications. First, 10,000 to 20,000 germ cells were bound to 10 µl concanavalin A-coated beads (Polysciences, 86057-10). After binding to beads, cells were fixed with 0.2% formaldehyde for 2 min followed by quenching with glycine (125 mM) and washed with Dig-Wash buffer while separated on the magnet. The remaining steps were as previously described^26^, using pA–Tn5 at a 1:400 dilution (Diagenode, C01070001) and 15 PCR cycles of library amplification. Libraries were cleaned up by magnetic bead-based solid-phase separation and assessed on a Tapestation (Agilent). Antibodies and dilutions used for CUT&Tag were rabbit IgG control (Abcam, ab37415, lot GR3219601-1) at 1:50, rabbit anti-SPIN1 (Cell Signaling, 89139S, lot 2) at 1:50, rabbit anti-H3K4me3 (Merck-Milipore, 07-473, lot 403371) at 1:50, rabbit anti-H3K9me3 (Abcam, ab8898, lot GR27111-1) at 1:50, and guinea pig anti-rabbit IgG (Antibodies Online, ABIN101961, lot NE-200-032309) at 1:100. Pooled libraries were sequenced using paired-end 150 bp on a NextSeq 2000 instrument (Illumina).

### CUT&Tag analysis

First, 150b and 155b paired-end CUT&Tag sequencing reads were processed and aligned to the mouse-genome assembly (version GRCm38) using the NF-core (10.5281/zenodo.7715959) CUT&RUN Nextflow pipeline version 3.1 (ref. ^53^). The pipeline performed adapter trimming with Trim Galore (10.5281/zenodo.5127898) and reference-genome alignment with Bowtie2 (ref. ^54^). Multimap reads were included using the parameter --minimum_alignment_q_score 0. The pipeline performed further filtering of reads to report only properly paired primary alignments and remove alignments to GRCm38 blacklisted regions. The default for the pipeline is to remove only duplicate reads (alignments that share common start and end points) from IgG controls. However, after further assessment of the sequence duplication rates in all samples, we decided to perform read deduplication on the SPIN1 replicate samples. Deduplication of SPIN1 samples was performed using Picard MarkDuplicates v.2.24.0 (http://broadinstitute.github.io/picard/) with the parameter --REMOVE_DUPLICATES. Individual replicates from each sample were then merged into a single BAM file using Samtools merge v.1.11 (ref. ^47^) for downstream analysis. Normalized bigWig files of read coverage were generated with deepTools bamCoverage v.3.50 (ref. ^48^), using the following parameters: -bs 1 --normalizeUsing CPM —exactScaling --ignoreForNormalization MT. Log_2_ enrichment profiles of CUT&Tag samples over IgG controls were generated with deepTools bamCompare using the following parameters: -bs 1 --normalizeUsing CPM --exactScaling --ignoreForNormalization MT --scaleFactorsMethod None.

Log_2_ enrichment profiles of CUT&Tag versus IgG control over various classes of repetitive elements (L1Md_A, L1Md_F, L1Md_Gf, L1Md_T, IAPEy-int and IAPEz-int) were plotted as heatmaps and average profiles, using computeMatrix from the deepTools^48^ package and the profilePlyr^55^ R package to include annotations of peak overlaps. Positions of repetitive elements were extracted from a table of mouse mm10 repeatMasker annotations downloaded from the UCSC table browser and filtered for elements greater than 5 kb in length. LINE1 elements (L1Md_A, L1Md_F, L1Md_T) were further separated into young LINE1 elements based on a divergence of 38 bases per kb or less from a consensus sequence^4^ or the presence of an intact functional promoter denoted by the presence of specific monomer annotations^49^. Monomers associated with inert promoters (subtypes 6 and 2) were removed from the analysis. The central regions of repetitive elements were length-normalized to 5 kb with flanking regions ±2 kb from the start and end positions. Heatmaps and profile plots show data in consecutive 10b bins with regions subdivided by elements and arranged in descending order of total enrichment across all samples.

Peak calling was done using MACS2 callpeak^56^ on individual replicates as well as all replicates together, with IgG samples set as a control. The parameter --keep-dup all was used to include duplicate reads, when present, in the peak calling model. To attain a set of high-confidence peaks, we selected peaks with a minimum coverage of 20 reads in the CUT&Tag sample and a peak score greater than the mean peak score. Peaks of co-localized H3K4me3 and H3K9me3 binding were attained by finding the intersection of both peak sets using the GenomicRanges R package^57^. Peak sets were overlapped with annotations to provide a breakdown of their intersection with specific genomic features, with each peak assigned to a single classification in the following hierarchy: LINEs, other repetitive elements, genes and intergenic. LINEs included all repeatMasker annotations included in the LINE class. Other repetitive elements included repeatMasker annotations in the classes LTR, Simple_repeat, Satellite, ERVK and Retrotransposon. Genes were defined as any coding or non-coding transcriptional unit plus 500 bases upstream, based on the ENSEMBL gene annotations GRCm38 v.79. Overlaps of peaks with genomic features was performed using the GenomicRanges R package^57^.

Downstream data analysis and plotting was predominantly performed using the R programming language (R Core Team, 2021, https://www.R-project.org/) and the Tidyverse libraries^51^. Genome snapshots and data tracks were prepared using pyGenomeTracks^58^.

### Histology of mouse samples

Histology experiments on mouse samples were done as previously described^4^.

### TUNEL assay

TUNEL assay experiments were done as previously described^4^.

### RNA sequencing and analysis

RNA sequencing experiments and analysis were done as previously described^4^ with data for *Spocd1*^−/−^ downloaded from GSE131377 (ref. ^4^).

### Whole-genome methylation sequencing and analysis

Whole-genome methylation sequencing of DNA derived from *Spocd1*^*ΔSPIN1*^ and wild-type P14 spermatogonia was performed using the NEBNext Enzymatic Methyl-seq (EM-seq, New England Biolabs) as described^4^. Analysis of DNA methylation was done as described previously^4^. Data for *Spocd1*^−/−^ and corresponding wild-type P14 spermatogonia were retrieved from E-MTAB-7997 (ref. ^4^).

### Statistical information

Data were plotted in R (v.2022.07.01 and 554 running R v.4.0.3 (2020-10-10)) using the dplyr, ggplot2, tidyr, cowplot, reshape2, ggrepel, ggpubr, scales and RColorBrewer packages (versions dplyr_1.0.4, ggplot2_3.3.3, tidyr_1.1.2, cowplot_1.1.1, scales_1.1.1, reshape2_1.4.4, ggrepel_0.9.1, ggpubr_0.4.0, scales_1.1.1, RColorBrewer_1.1-2) or Microsoft Excel for Mac (v.16). Statistical testing was done with R (v.4.0.3 (2020-10-10)) using R Studio software or with Perseus^59^ (v.1.6.5.0) for the mass-spectrometry data and DEseq2 (ref. ^60^) for the RNA-seq data. We used the regioneR package^55^ in R to perform permutation tests to assess the statistical significance of overlaps of CUT&Tag peaks with LINE1 elements. Unpaired, two-tailed Student’s *t*-tests were used to compare the differences between groups and adjusted for multiple testing using Bonferroni correction where indicated, except for RNA-seq data analysis, where Wald’s tests were used. Averaged data are presented as mean ± s.e.m., unless otherwise indicated. No statistical methods were used to predetermine the sample size. The experiments were not randomized and the investigators were not blinded to allocation during experiments and outcome assessment.

### Reporting summary

Further information on research design is available in the Nature Portfolio Reporting Summary linked to this article.

## Online content

Any methods, additional references, Nature Portfolio reporting summaries, source data, extended data, supplementary information, acknowledgements, peer review information; details of author contributions and competing interests; and statements of data and code availability are available at 10.1038/s41586-024-07963-3.

## Supplementary information

Supplementary Figures Supplementary Figures containing uncropped scans of the western-blot experiments shown in Figs. 1d–g,j and 4a, and Extended Data Fig. 5a (Supplementary Fig. 1), and the FACS gating strategy for sorting fetal germ cells and undifferentiated spermatogonia (Supplementary Fig. 2).

Reporting Summary

Supplementary Table 1 Further data relating to crosslinking mass spectrometry data.

## Figures and Tables

**Extended Data Table 1:**
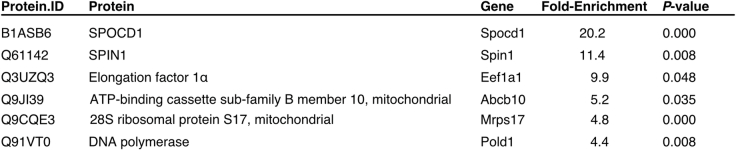
Proteins identified as SPOCD1 interactors in E14.5 foetal testes

Table listing all statistically significant (*P* < 0.05, two-sided Student’s *t*-test, *n* = 3) proteins that are at least 4-fold enriched in the SPOCD1-HA immuno-precipitation.

## Data Availability

The EM-seq data generated in this study have been deposited on ArrayExpress under accession number E-MTAB-12713. The RNA-seq data generated in this study have been deposited at the Gene Expression Omnibus under GSE228294 and the CUT&Tag data generated here are at GSE269344. The data for the IP-MS experiment have been deposited at ProteomeXchange under the accession number PXD041214 and the crosslink MS data are under PXD041135. The publicly available datasets used in this study are the ChIP-seq of H3K4me3 and H4K9me3, downloaded from the Sequence Read Archive record SRP165187; the RNA-seq data for *Spocd1*^−/−^, downloaded from GSE131377; and the EM-seq data for *Spocd1*^−/−^ and corresponding wild-type P14 spermatogonia, retrieved from E-MTAB-7997 (https://www.ebi.ac.uk/biostudies/arrayexpress/studies/E-MTAB-7997).

## Code availability

The scripts used for the EM-seq and RNA-seq analysis are available from github at https://github.com/rberrens/SPOCD1-piRNA_directed_DNA_met, and the scripts used for ChIP and CUT&Tag analysis are available from github at https://github.com/swebb1/heep-et-al_2024.
