# Supplementary material for: Two-factor authentication underpins the precision of the piRNA pathway
Source: Nature. 2024 Sep 18;634(8035):979–85. doi: 10.1038/s41586-024-07963-3 (PMC11499256; doi:10.1038/s41586-024-07963-3)
Supplement: Supplementary file 1 — Supplementary Figures containing uncropped scans of the western-blot experiments shown in Figs. 1d–g,j and 4a, and Extended Data Fig. 5a (Supplementary Fig. 1), and the FACS gating strategy for sorting fetal germ cells and undifferentiated spermatogonia (Supplementary Fig. 2). [file 41586_2024_7963_MOESM1_ESM.pdf]

nature portfolio
https://doi.org/10.1038/s41586-024-07963-3

Supplementary information

Two-factor authentication underpins the precision of the piRNA pathway

In the format provided by the authors and unedited

Supplemental Data Figure - Whole blot images of western blot figures

Whole blots used for Figure 1 d

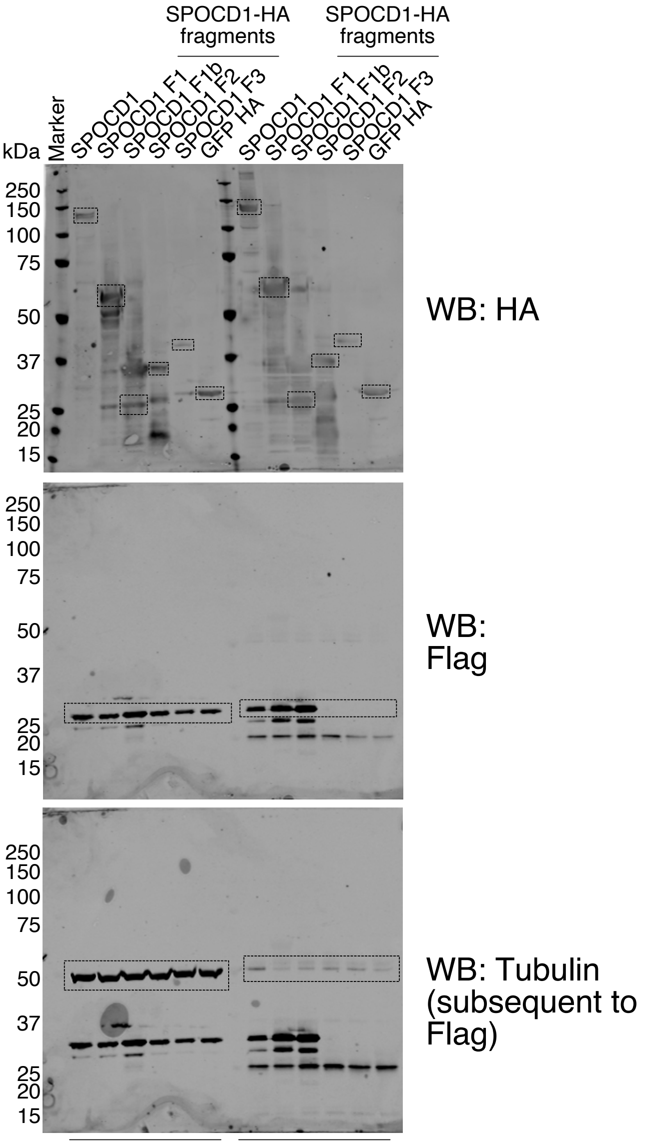

indicates cropped imaged shown in figure

Whole blots used for Figure 1 g

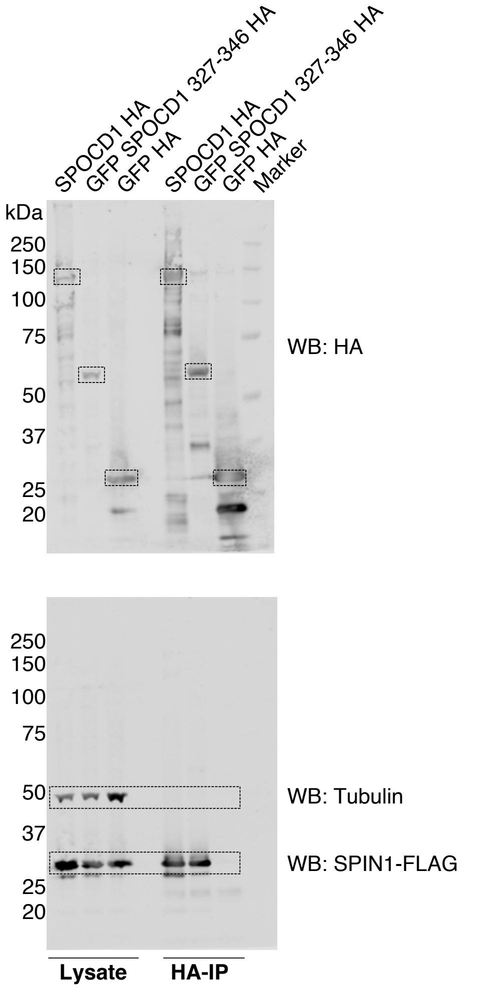

indicates cropped imaged shown in figure

Whole blots used for Figure 1 e

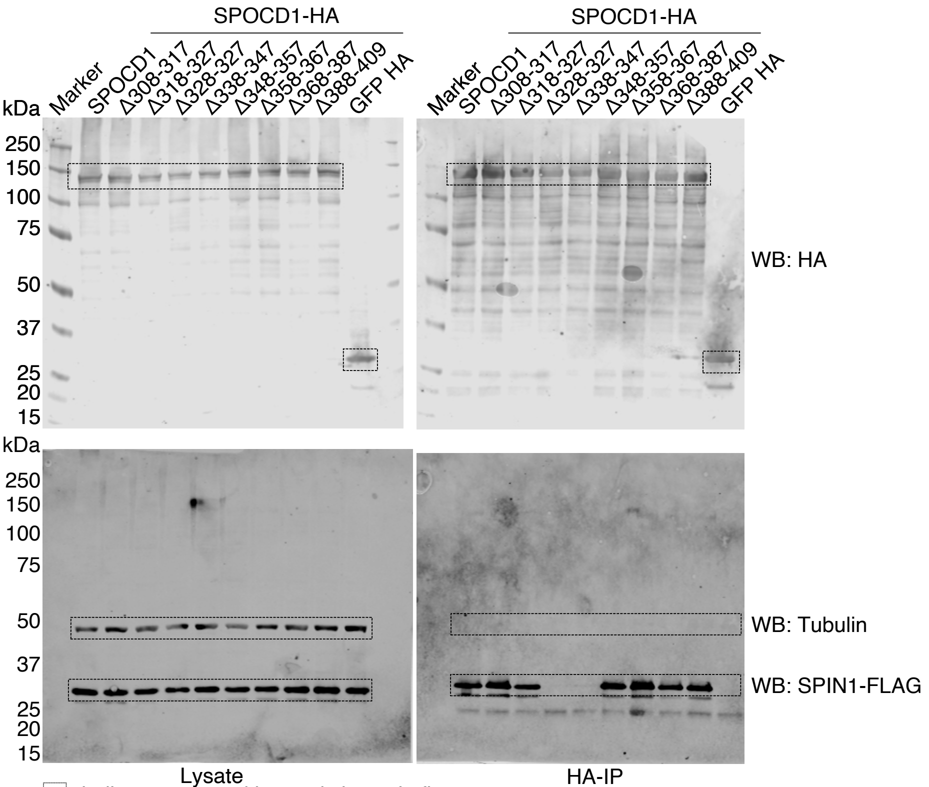

indicates cropped imaged shown in figure

Supplemental Data Figure - Whole blot images of western blot figures

Whole blots and gels used for Figure 1 j

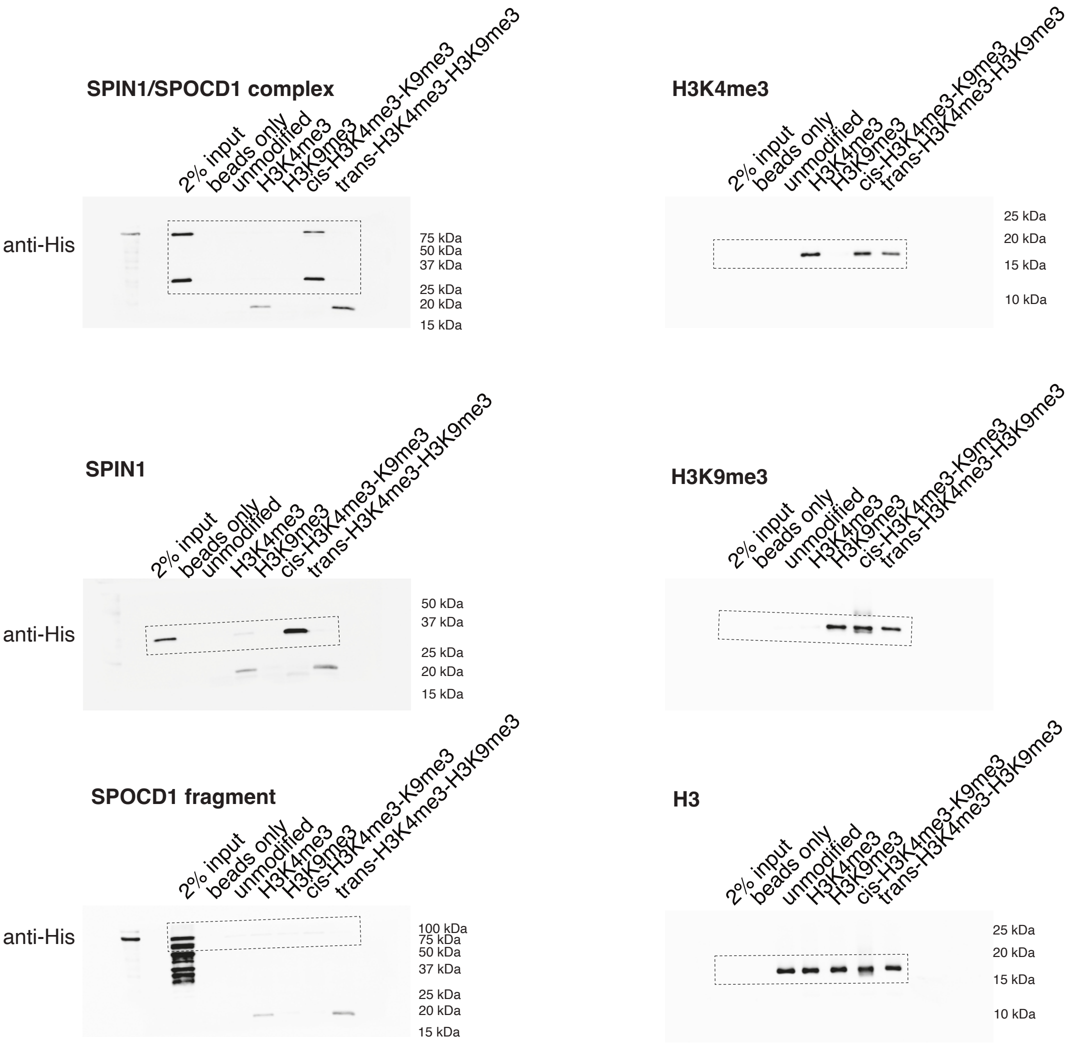

* 75 kDa marker band cross-reacts with anti-His antibody

indicates cropped imaged shown in figure

Supplemental Data Figure - Whole blot images of western blot figures

Whole blots used for Figure 4 a

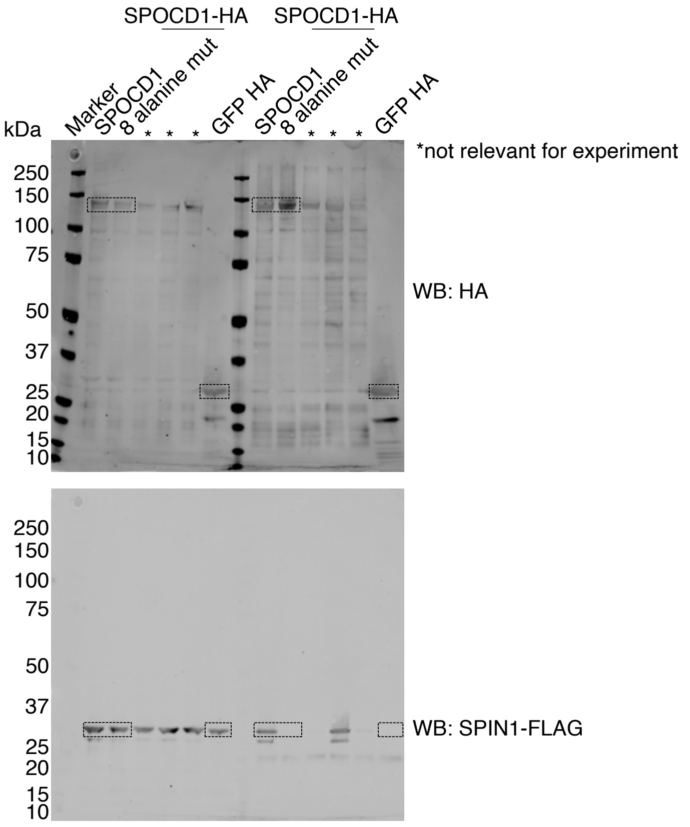

☐ indicates cropped imaged shown in figure

Whole blots used for Extended Data Figure 5 a

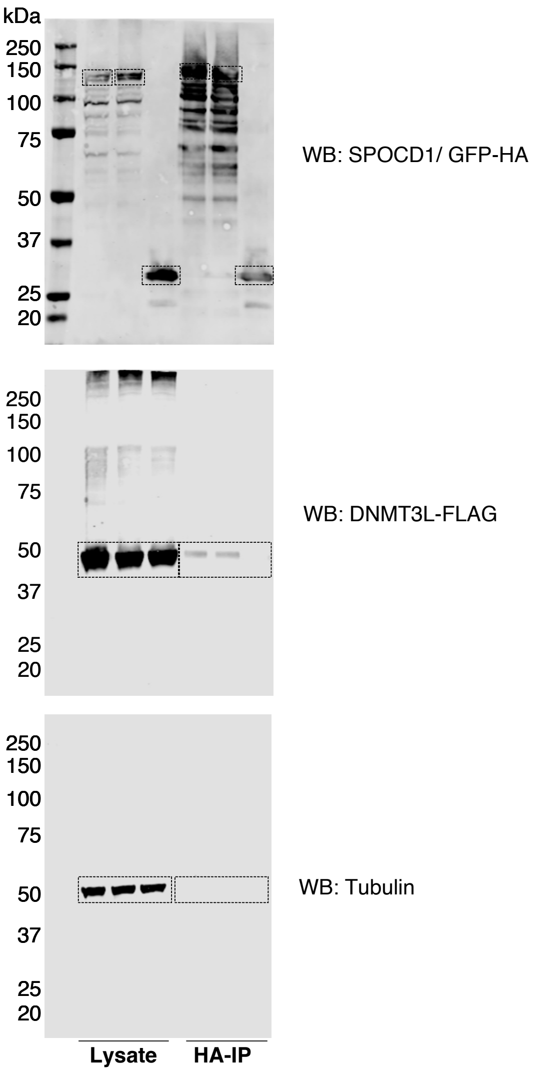

☐ indicates cropped imaged shown in figure

Supplementary Figure - FACS gating

a

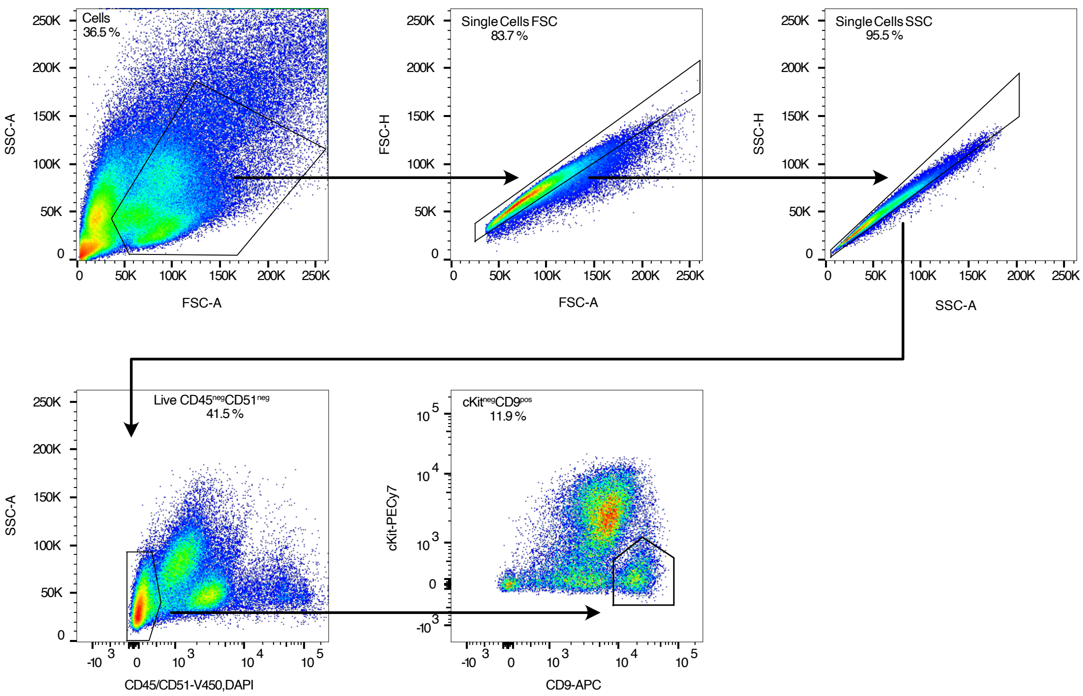

b

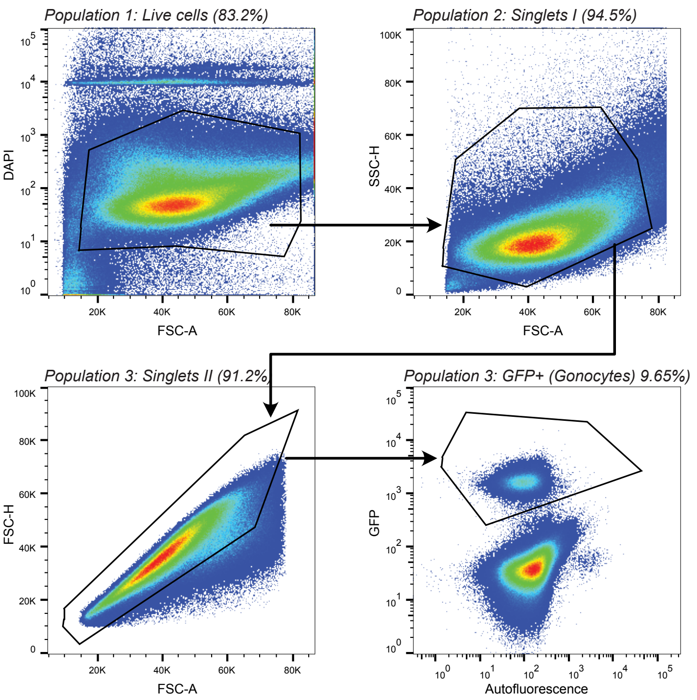

Supplemental Figure – Gating strategy for sorting P14 spermatogonia and E14.5 gonocytes

a, Gating strategy to sort P14 CD9$^+$ spermatogonia. **b,** Representative FACS gating strategy to isolate live gonocytes from single cell suspensions of embryonic testes using the Oct4-EGFP reporter (B6;129S4-Pou5f1^{tm2Jae}/J).
