# Supplementary material for: Two-factor authentication underpins the precision of the piRNA pathway
Source: Nature. 2024 Sep 18;634(8035):979–85. doi: 10.1038/s41586-024-07963-3 (PMC11499256; doi:10.1038/s41586-024-07963-3)
Supplement: Supplementary file 2 — Reporting Summary [file 41586_2024_7963_MOESM2_ESM.pdf]

Corresponding author(s): Donal O'Carroll
Last updated by author(s): Jul 24, 2024

Reporting Summary

Nature Portfolio wishes to improve the reproducibility of the work that we publish. This form provides structure for consistency and transparency in reporting. For further information on Nature Portfolio policies, see our Editorial Policies and the Editorial Policy Checklist.

Statistics

For all statistical analyses, confirm that the following items are present in the figure legend, table legend, main text, or Methods section.

n/a	Confirmed	
☐	☒	The exact sample size (*n*) for each experimental group/condition, given as a discrete number and unit of measurement
☐	☒	A statement on whether measurements were taken from distinct samples or whether the same sample was measured repeatedly
☐	☒	The statistical test(s) used AND whether they are one- or two-sided<br>*Only common tests should be described solely by name; describe more complex techniques in the Methods section.*
☒	☐	A description of all covariates tested
☐	☒	A description of any assumptions or corrections, such as tests of normality and adjustment for multiple comparisons
☐	☒	A full description of the statistical parameters including central tendency (e.g. means) or other basic estimates (e.g. regression coefficient) AND variation (e.g. standard deviation) or associated estimates of uncertainty (e.g. confidence intervals)
☐	☒	For null hypothesis testing, the test statistic (e.g. *F*, *t*, *r*) with confidence intervals, effect sizes, degrees of freedom and *P* value noted<br>*Give P values as exact values whenever suitable.*
☒	☐	For Bayesian analysis, information on the choice of priors and Markov chain Monte Carlo settings
☒	☐	For hierarchical and complex designs, identification of the appropriate level for tests and full reporting of outcomes
☐	☒	Estimates of effect sizes (e.g. Cohen's *d*, Pearson's *r*), indicating how they were calculated

Our web collection on statistics for biologists contains articles on many of the points above.

Software and code

Policy information about availability of computer code

Data collection	Microscope images were acquired using Zeiss Zen Black (2.3 SP1) and Zen Blue (3.5) software as well as Leica Application Suite (LAS-X 3.5.5) software. BF FACS DIVA (8.0.1) and Invitrogen Sasquatch v1.19.4 software were used for cell sorting.
Data analysis	The software used to analyze the data is described in the respective methods sections and the scripts used to analyze data and generate graphs have been made available at https://github.com/rberrens/SPOCD1-piRNA_directed_DNA_met and https://github.com/swebb1/heep-et-al_2024.<br>Software versions used:<br><br>Image analysis:<br>ImageJ2 1.54f as part of the FIJI package.<br><br>ChIP-seq analysis software:<br>Trimmomatic v0.35<br>bwa v0.7.16<br>Picard v2.24.0<br>Samtools v1.11<br>deepTools v3.5.0<br><br>CUT&RUN analysis software:<br>CUT&RUN Nextflow pipeline v3.1<br>Picard v2.24.0

Samtools v1.11
deepTools v3.5.0
MACS2 2.2.7.1
pyGenomeTracks 3.9

R statistics and plotting packages:
R v4.03
dplyr_1.0.4
ggplot2_3.3.3
tidyr_1.1.2
cowplot_1.1.1
scales_1.1.1
reshape2_1.4.4
ggrepel_0.9.1
ggpubr_0.4.0
scales_1.1.1
RColorBrewer_1.1-2
regioneR _1.3
GenomicRanges_1.5.2
DESeq2_1.38.3

Mass spectrometry analysis:
Perseus v1.6.5.0

For manuscripts utilizing custom algorithms or software that are central to the research but not yet described in published literature, software must be made available to editors and reviewers. We strongly encourage code deposition in a community repository (e.g. GitHub). See the Nature Portfolio guidelines for submitting code & software for further information.

Data

Policy information about availability of data

All manuscripts must include a data availability statement. This statement should provide the following information, where applicable:

- Accession codes, unique identifiers, or web links for publicly available datasets
- A description of any restrictions on data availability
- For clinical datasets or third party data, please ensure that the statement adheres to our policy

The EM-seq data generated in this study have been deposited on ArrayExpress under accession number E-MTAB-12713. The RNA-seq data generated in this study has been deposited at the Gene Expression Omnibus under GSE228294. CUT&Tag data generated in this study has been deposited at GSE269344. Data for the IP-MS experiment was deposited at ProteomeXchange under the accession number PXD041214. The crosslink MS data is deposited under PXD041135.
Additional datasets used in this study: mouse genome (GRCm38), Spocd1-null EM-seq (Arrayexpress E-MTAB-7997) and RNA-seq (GEO GSE131377) datasets, ChIP-seq dataset (Sequence Read Archive record SRP165187), repeat annotation (UCSC repeatmasker track).

Research involving human participants, their data, or biological material

Policy information about studies with human participants or human data. See also policy information about sex, gender (identity/presentation), and sexual orientation and race, ethnicity and racism.

Reporting on sex and gender	N/A
Reporting on race, ethnicity, or other socially relevant groupings	N/A
Population characteristics	N/A
Recruitment	N/A
Ethics oversight	N/A

Note that full information on the approval of the study protocol must also be provided in the manuscript.

Field-specific reporting

Please select the one below that is the best fit for your research. If you are not sure, read the appropriate sections before making your selection.

☒ Life sciences ☐ Behavioural & social sciences ☐ Ecological, evolutionary & environmental sciences

For a reference copy of the document with all sections, see nature.com/documents/nr-reporting-summary-flat.pdf

Life sciences study design

All studies must disclose on these points even when the disclosure is negative.

Sample size	No statistical methods were used to predetermine the sample size. Based on the biological material available, the sample size was determined. For each experiment, three or more biological replicates were analysed to sufficiently calculate statistics.
Data exclusions	No datasets were excluded from the analysis.
Replication	All mouse experimental data shown has been generated from biological replicates which showed consistent outcomes within each group for all experiments. For all experiments at least three independent biological replicates were used, except for CUT&Tag of H3K4me3 and H4K9me3 with used two replicates. The exact sample size is noted in each figure legend. IP-WB experiments in HEK cells and co-precipitation with recombinant proteins were repeated three times with similar results. Analytical size exclusion and crosslink mass-spectrometry was repeated two times with similar results.
Randomization	Samples were allocated to groups according to genotype. Experimental and control samples were processed in parallel.
Blinding	No blinding was used during data acquisition and analysis due to the small number of samples processed at any one time.

Reporting for specific materials, systems and methods

We require information from authors about some types of materials, experimental systems and methods used in many studies. Here, indicate whether each material, system or method listed is relevant to your study. If you are not sure if a list item applies to your research, read the appropriate section before selecting a response.

Materials & experimental systems

n/a	Involved in the study	
☐	☒	Antibodies
☐	☒	Eukaryotic cell lines
☒	☐	Palaeontology and archaeology
☐	☒	Animals and other organisms
☒	☐	Clinical data
☒	☐	Dual use research of concern
☒	☐	Plants

Methods

n/a	Involved in the study	
☒	☐	ChIP-seq
☐	☒	Flow cytometry
☒	☐	MRI-based neuroimaging

Antibodies

Antibodies used	anti-HA (Cell Signaling Technologies, 3724, C29F4, IF: 1:200, WB: 1:1000, lot 12); anti-FLAG (Sigma-Aldrich, F1804, M2, WB: 1:1000, lot 261540), anti-SPOCD1 rabbit serum rb175 (O'Carroll laboratory antibody, IF: 1:500, WB: 1:500), anti-α-Tubulin (Sigma-Aldrich, T9026, WB: 1:10,000, lot 137585), anti-HA (Cell Signaling Technologies, 2367, 6E2, IF: 1:200, lot 5), anti-LINE1-ORF1p (di Giacomo et al, 2014, IF: 1:500), anti-IAP-GAG (a kind gift from B. Cullen, Duke University, Durham, NC, USA, IF: 1:500), anti-γH2AX (Bethyl Laboratories, IHC-00059, IF: 1:500, lot 5), anti-MIWI2 (a kind gift from Ramesh Pillai, Université de Genève, Switzerland IF: 1:500), anti-SPIN1 (Cell Signaling Technologies, 89139S, E6R1Z, IF: 1: 500 and CUT&Tag 1:50 (of a custom preparation of 1.1 µg/µl in PBS), lot 2), anti-His tag (Sigma, H1029, WB 1:1000, lot 033m4785), anti-histone H3 (Abcam, ab176842, WB 1:2500, lot GR1494741-36), anti-H3K4me3 (Cell Signaling, 9733, WB 1:2000, lot 14), anti-H3K9me3 (Abcam, ab176916, WB 1:1000, lot GR3218257-13), rabbit IgG control (Abcam, ab37415, CUT&Tag 1:50, lot GR3219601-1), rabbit anti-H3K4me3 (Merck-Milipore, 07-473, CUT & Tag 1:50, lot 403371), rabbit anti-H3K9me3 (Abcam, ab8898, CUT&Tag 1:50, lot GR27111-1), anti-SPIN1 (Cell Signaling Technologies, 89139S, E6R1Z, CUT&Tag 1:50, lot 1), and guinea pig anti-rabbit IgG (Antibodies online, ABIN101961, CUT & Tag 1:100, lot NE-200-032309), anti-CD16/32 (eBioscience, 14-0161-86, clone 93, FACS: 1:50, lot 2297433), anti-CD45 (eBioscience, 13-0451-85, clone 30-F11, FACS: 1:400, lot 2349865), anti-CD51 (Biolegend, 104104, clone RMV-7, FACS: 1:100, lot B308465), anti-CD9-APC (eBioscience, 17-0091-82, clone eBioKMC8, FACS: 1:200, lot 2450733), anti-cKit-PE-Cy7 (eBioscience, 25-1171-82, clone 2B8, FACS: 1:1600, lot 2191977), streptavidinV450 (BD bioscience, 560797, FACS: 1:400, lot. 1354158), anti-HA beads (Pierce, 88837, IP-MS: 50 µl, IP-WB: 20 µl, lot YD360611).
Validation	The anti-HA antibodies were validated and used for IF and WB previously (Zoch et al. 2020). The anti-FLAG antibody is a widely used antibody and was used for WB previously (Zoch et al. 2020). The specificity of anti-SPOCD1 rabbit serum rb175 was validated for IF by staining of Spocd1+/+ and Spocd1-/- E16.5 foetal testis sections; and was validated for WB by probing of extracts with and without SPOCD1. The anti-α-Tubulin antibody is widely used and was validated for WB using recombinant protein samples. The anti-LINE1-ORF1p (described in di Giacomo et al. 2014) and anti-IAP-GAG (described in Mietz et al. 1987) have been validated for IF on mouse sections previously. The anti-γH2AX antibody has been used previously for IF on mouse sections (Vasiliauskaite et al. 2018, Much et al. 2016, Comazzetto et al. 2014, Zoch et al. 2020). The anti-MIWI2 antibody has been validated for IF on mouse sections previously (Pandey et al. 2013). The anti-CD16/32, anti-CD45, anti-CD51, anti-CD9 and anti-cKit antibodies have been validated for FACS of undifferentiated spermatogonia before (Vasiliauskaite et al. 2017, Zoch et al. 2020). The anti-HA beads have been used for IP in previous studies (Zoch et al. 2020).

Eukaryotic cell lines

Policy information about cell lines and Sex and Gender in Research

Cell line source(s)	HEK 293T: sourced from O'Carroll laboratory (University of Edinburgh). HEK 293T cells can be sourced form ATCC product (CRL-3216) (https://www.atcc.org/products/crl-3216).
Authentication	Cell lines were not authenticated.
Mycoplasma contamination	Cells were routinely tested negative for mycoplasma contamination.
Commonly misidentified lines (See ICLAC register)	No commonly misidentified cell lines were used in the study.

Animals and other research organisms

Policy information about studies involving animals; ARRIVE guidelines recommended for reporting animal research, and Sex and Gender in Research

Laboratory animals	Mice:<br>Spocd1-HA (B6CBAF1/Crl;C57BL/6N;Hsd:ICR (CD1)): male embryo E14.5 and E16.5<br>Miwi2-tdTomato (B6CBAF1/Crl;C57BL/6N;Hsd:ICR (CD1)): male embryo E16.5<br>Spocd1-dSpin1 (B6CBAF1/Crl;C57BL/6N): male embryo E16.5, males postnatal day 14, males postnatal day 20, male and female 8-16 weeks<br>Hsd:ICR (CD1): males E14.5, males and females 8-16 weeks<br>Oct4-eGFP (Jackson Laboratories: B6;129S4-Pou5f1tm2Jae/J (Oct4-eGFP)): male embryo E14.5<br><br>Mice were housed under the following conditions:<br>Lighting : 12 hour light to dark cycle 7am to 7pm<br>Temperature: 20-24 oC<br>Humidity: 45-65 %<br>Caging : Techniplast GM 500 Individual ventilated cages<br>Cage substrate: aspen chips<br>Enrichment: aspen chew sticks, carboard dome home, rodent roll and plastic tube for non-aversive handling.
Wild animals	This study does not involve wild animals.
Reporting on sex	This study only analyses male mice because female mice with defects in the piRNA pathway are fertile and do not show any phenotype.
Field-collected samples	This study does not involve samples collected from the field.
Ethics oversight	Ethical approval for the mouse experimentation has been given by the University of Edinburgh's Animal Welfare and Ethical Review Body and the work done under licence from the United Kingdom's Home Office as well as ethical committees on animal care and use of the federal states of Rheinland-Pfalz, Germany.

Note that full information on the approval of the study protocol must also be provided in the manuscript.

Flow Cytometry

Plots

Confirm that:

- [x] The axis labels state the marker and fluorochrome used (e.g. CD4-FITC).
- [x] The axis scales are clearly visible. Include numbers along axes only for bottom left plot of group (a 'group' is an analysis of identical markers).
- [x] All plots are contour plots with outliers or pseudocolor plots.
- [x] A numerical value for number of cells or percentage (with statistics) is provided.

Methodology

Sample preparation	The sample preparation for P14 spermatogonia was done as described previously (Vasiliauskaite et al. 2018). Briefly, P14 testes were deglutinated and digested with collagenase followed by Trypsin and DNase digest. Single cells were labelled with anti-CD16/32, anti-CD45, anti-51, anti-cKit and anti-CD9 antibodies.<br>Gonocyte sample preparation was done from E14.5 foetal Oct4eGFP/+ testes by successive digestion with collagenase and TryPLE Express (Gibco). DAPI was added to single cell suspension to distinguish live cells.

Instrument	BD Aria II, BD Aria II Fusion and Invitrogen Bigfoot
Software	BD FACS Diva (8.0.1), FloJo8, Invitrogen Sasquatch
Cell population abundance	P14 undifferentiated CD9+, cKit- spermatognia were 98% pure (see Vasiliauskaite et al. 2017), Oct4-eGFP positive E14.5 gonocytes were 99% pure.
Gating strategy	The gating strategy of P14 spermatogonia has been described previously (Vasilauskaite et al. 2018): live cells were gated for CD51/45 negative cells and sorted as CD9 high, cKit negative population. Gates were set based on fluorescence intensity in the fully stained sample minus one sample (lacking the label for the marker of interest) for each label. E14.5 gonocytes sorting was done by gating for live DAPI negative and singlet cells and sorted as an Oct4-eGFP positive population.

☒ Tick this box to confirm that a figure exemplifying the gating strategy is provided in the Supplementary Information.
